# hnRNPC Functions with HuR to Regulate Alternative Splicing in an m6A‐Dependent Manner and is Essential for Meiosis

**DOI:** 10.1002/advs.202412196

**Published:** 2025-02-08

**Authors:** Xinxin Xiong, Shenglei Feng, Xixiang Ma, Kuan Liu, Yiqian Gui, Bei Chen, Xu Fan, Fengli Wang, Xiaoli Wang, Shuiqiao Yuan

**Affiliations:** ^1^ Institute of Reproductive Health, Tongji Medical College Huazhong University of Science and Technology Wuhan 430030 China; ^2^ Laboratory Animal Center Huazhong University of Science and Technology Wuhan 430030 China; ^3^ Reproductive Medicine Center Renmin Hospital of Wuhan University Wuhan 430060 China; ^4^ Shenzhen Huazhong University of Science and Technology Research Institute Shenzhen 518057 China

**Keywords:** germ cells, hnRNPC, HuR, m6A modification, meiosis

## Abstract

N6‐methyladenosine (m6A) and its reader proteins are involved in pre‐mRNA processing and play a variety of roles in numerous biological processes. However, much remains to be understood about the regulation of m6A and the function of its specific readers during meiotic processes. Here, this study shows that the potential m6A reader protein hnRNPC is essential for both male and female meiosis in mice. Germ cell‐specific knockout of *Hnrnpc* causes meiotic arrest at pachynema in male mice. Specifically, hnRNPC‐deficient males show abnormal meiosis initiation and defective meiotic progression, ultimately leading to meiotic arrest at the pachytene stage. Interestingly, hnRNPC‐null females show similar meiotic defects to males. Mechanistically, this study discovers that in male germ cells, hnRNPC works with HuR to directly bind and modulate alternative splicing of meiotic‐related genes (e.g., *Sycp1*, *Brca1*, and *Smc5*) in an m6A‐dependent manner during spermatogenesis. Collectively, these findings reveal hnRNPC as a critical factor for meiosis and contribute to a mechanistic understanding of the hnRNPC‐HuR interaction in alternative splicing of mRNAs during germ cell development.

## Graphical abstract

This work shows that hnRNPC recognizes and binds directly to key meiosis‐related genes and works with HuR to regulate alternative splicing of its target genes, ensuring the normal production and proper function of essential meiotic transcripts required for meiotic initiation and progression during the first wave of spermatogenesis.

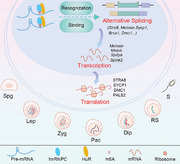

## 1 Introduction

Mammalian spermatogenesis is a complex and orchestrated process that produces haploid spermatozoa. In adult male mice, spermatogonial stem cells (SSCs) undergo mitotic expansions to produce A‐paired (Apr) and A‐aligned (Aal) spermatogonia, and subsequently differentiate into different types of spermatogonia, eventually entering meiotic prophase.^[^ ^1^ ^]^ At the mitosis‐to‐meiosis transition, MEIOSIN, a germ cell‐specific transcription factor, works together with STRA8 to direct the initiation of meiosis.^[^ ^2^ ^]^ Programmed double‐strand breaks (DSBs) are then generated during the leptotene stage and are catalyzed by the SPO11‐TOPOVIBL complex and pre‐DSB recombinases (e.g., REC114, MEI1, MEI4, ANKRD31, and IHO1).^[^ ^3^ ^]^ Subsequently, DSB repair is completed with concurrent crossover formation before late pachytene.^[^ ^4^ ^]^ After two consecutive meiotic divisions, haploid round spermatids are produced and undergo morphological changes to transform into mature spermatozoa. Each step of spermatogenesis is orchestrated by multiple factors at both transcriptional and post‐transcriptional levels,^[^ ^5^, ^6^ ^]^ and any defect in these factors could affect spermatogenesis.

N6‐methyladenosine (m6A) is the most abundant mRNA internal modification in mammals and is present on almost all types of RNA involved in diverse fundamental cellular processes,^[^ ^7^, ^8^ ^]^ including pre‐mRNA splicing, mRNA stability, and translation. m6A is installed by methyltransferases (writers), such as METTL3/14/16 and WTAP,^[^ ^9^, ^10^, ^11^ ^]^ removed by demethylases (erasers), such as FTO and ALKBH5,^[^ ^12^ ^]^ and recognized by “readers”, including the YTH family,^[^ ^13^ ^]^ IGF2 mRNA binding proteins (IGF2BPs),^[^ ^14^ ^]^ PRRC2A,^[^ ^15^ ^]^ and several members of the heterogeneous nuclear ribonucleoproteins (HNRNPs) family.^[^ ^16^, ^17^ ^]^ In particular, accumulating evidence has shown that the m6A modification is essential for germ cell development and spermatogenesis. Conditional knockout of *Mettl3* or *Mettl14* results in the depletion of SSCs.^[^ ^18^ ^]^ Mice deficient in *Alkbh5* exhibit defects with apoptosis of pachytene and metaphase spermatocytes and aberrant spermiogenesis.^[^ ^19^ ^]^ In addition, PRRC2A as an m6A reader has been reported to exert its post‐transcriptional functions in meiosis I during spermatogenesis,^[^ ^20^ ^]^ and the conditional knockout of another m6A reader protein, YTHDF2, resulted in oligoasthenoteratozoospermia in mice.^[^ ^21^ ^]^ However, it is unclear whether HNRNPs are involved in spermatogenesis as m6A reader proteins.

To date, three hnRNP members have been identified as potential m6A readers, including hnRNPA2B1,^[^ ^22^ ^]^ hnRNPG,^[^ ^23^ ^]^ and hnRNPC.^[^ ^24^ ^]^ hnRNPC binds m6A‐modified RNAs through an “m6A switch” mechanism in which m6A modification alters RNA structure and exposes a U‐rich binding site for hnRNPC to regulate gene expression and maturation.^[^ ^24^ ^]^ Dysfunction of hnRNPC has been implicated in various diseases including autoimmune thyroid disease, breast cancer, and pancreatic ductal adenocarcinoma.^[^ ^25^, ^26^, ^27^ ^]^ Notably, our previous study found that hnRNPC in Sertoli cells plays a crucial role in supporting normal spermatogenesis,^[^ ^28^ ^]^ but the function of hnRNPC in germ cells remains unknown. In this study, we have shown that hnRNPC is highly expressed in spermatogenic cells, particularly spermatogonia and spermatocytes. Conditional knockout of hnRNPC in germ cells resulted in infertility in both male and female mice. In males, hnRNPC ablation impaired meiotic initiation and progression, ultimately leading to meiotic arrest at the pachytene stage; similarly, female mice phenocopied the meiotic defects in males upon hnRNPC deletion. In addition, we found that hnRNPC acts as an m6A reader and regulates alternative splicing depending on the m6A modification in differentiated spermatogonia. We further found that hnRNPC can synergize with HuR, an RNA‐binding protein belonging to the ELAV (*embryonic lethal abnormal visual system*) family, which has been documented to be involved in the regulation of alternative splicing^[^ ^29^, ^30^, ^31^ ^]^ and male meiotic progression,^[^ ^32^ ^]^ to modulate alternative splicing of meiotic‐related genes during spermatogenesis. Taken together, our results reveal an essential function of hnRNPC in germ cells and demonstrate that hnRNPC regulates alternative splicing by cooperating with HuR during germ cell development.

## 2 Results

### 2.1 hnRNPC Deficiency Leads to Male Sterility in Mice

**Figure 1:**
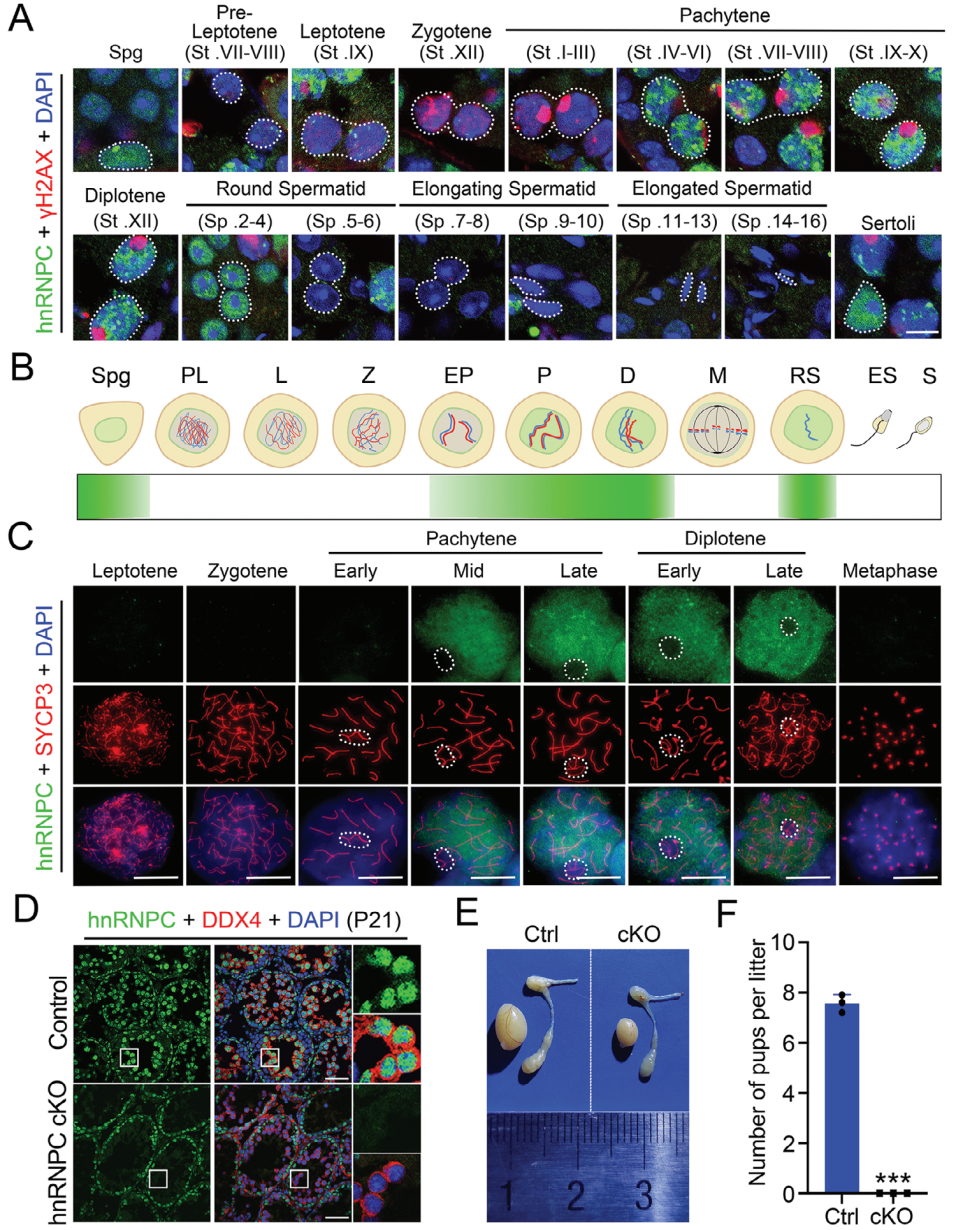
hnRNPC displays a dynamic expression pattern during spermatogenesis and is essential for male fertility. A) Representative confocal immunofluorescence (IF) images with anti‐hnRNPC antibody (green), anti‐γH2AX antibody (red), and DAPI (blue) on testis sections from 8‐week‐old WT mice. White dotted lines indicate specific cell type. Scale bar = 10 µm. B) Schematic diagram of hnRNPC expression during spermatogenesis. Spg: spermatogonia; PL, pre‐leptotene; L, leptotene; Z, zygotene; EP, early pachytene; P, pachytene; D, diplotene; M, metaphase; RS, round spermatids; ES, elongating spermatids; S, spermatozoa. C) Immunostaining with anti‐hnRNPC antibody (green), anti‐γH2AX antibody (red), and DAPI (blue) on nuclear spreading spermatocytes from adult WT mice. White circles indicate XY bodies. Scale bars = 5 µm. D) Representative confocal images of IF with anti‐hnRNPC antibody (green), anti‐DDX4 antibody (red), and DAPI (blue) on testis sections from control and hnRNPC cKO (cKO) mice at postnatal day 21 (P21). Scale bars = 50 µm. E) Morphological analysis of testes and epididymides of adult control and hnRNPC cKO mice. F) Fertility ability of control and cKO male mice (n = 3 per group) mated with WT female mice. Error bars represent mean ± SD. Two‐sided Student's *t*‐test. ^***^ *p *<0.001.

To test whether hnRNPC carries an important role in male germ cells, we first examined its expression pattern and subcelluar localization by immunofluorescence (IF) assay on testis sections from adult wild‐type (WT) mice. hnRNPC showed prominent expression in the nuclei of spermatogonia, pachytene spermatocytes, diplotene spermatocytes, early round spermatids (until step 4), and Sertoli cells (**Figure** **1**A,B). IF on chromosome spreads further demonstrated that hnRNPC was first detectable from mid‐pachytene and persisted until diplotene spermatocytes but was excluded from the XY body (Figure 1C). Consistently, the colocalization of hnRNPC and H1T (a specific maker of spermatocytes from mid‐pachytene onward^[^ ^33^ ^]^) in postnatal day 14 (P14) testes confirmed its predominant expression in mid‐pachytene spermatocytes (Figure S1A, Supporting Information); as for the spermatogonia, strong signals of hnRNPC were observed in both the undifferentiated (SALL4 or PLZF positive) and differentiated (c‐KIT positive) spermatogonia (Figure S1B–D, Supporting Information).

To investigate the physiological role of hnRNPC in spermatogenesis, we crossed *Hnrnpc* ^flox/flox^ mice with the exon 4–5 flanked by loxP sites with *Stra8‐GFP*Cre mice,^[^ ^18^ ^]^ which begin to express Cre recombinase in the testis from P3 to generate a germ cell‐specific *Hnrnpc* knockout mouse model (*Stra8‐GFP*Cre; *Hnrnpc* ^flox/del^, referred to as hnRNPC cKO or cKO) (Figure S2A, Supporting Information). The genotype was analyzed by PCR and the successful deletion of hnRNPC was confirmed by RT‐qPCR, Western blot (WB), and IF, respectively (Figure 1D; Figure S2B–F, Supporting Information). hnRNPC cKO males had normal body weight and appeared healthy, but their testes were smaller and they did not produce any offspring (Figure 1E,F; Figure S2G, Supporting Information). Together, these results suggest that hnRNPC is highly expressed in male germ cells and is essential for male fertility in mice.

### 2.2 Delayed Meiosis Occurred in hnRNPC cKO Testes

**Figure 2:**
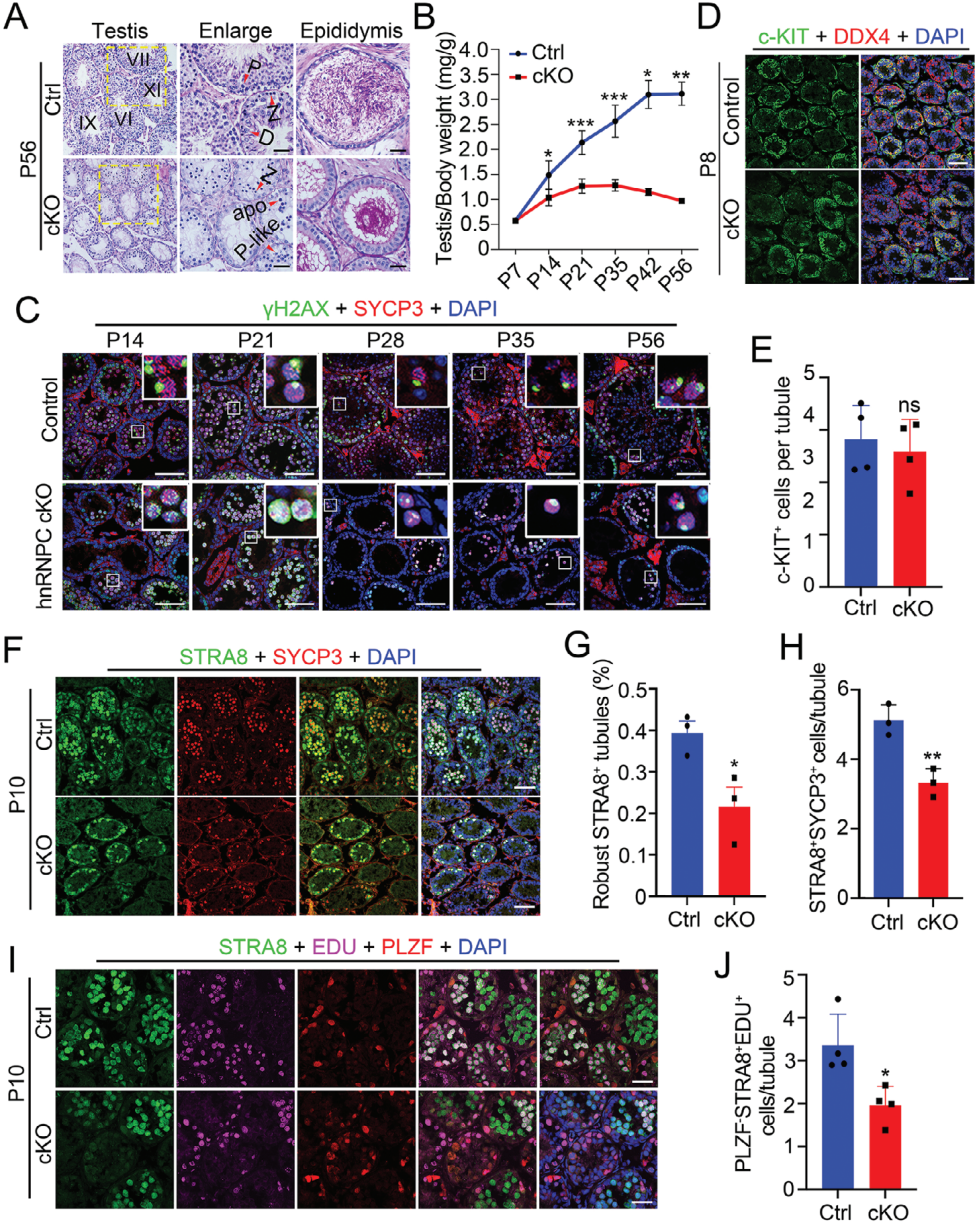
hnRNPC deficiency leads to delayed meiosis. A) Periodic acid‐Schiff (PAS) staining of WT and hnRNPC cKO testis and epididymis sections at P56. Z, zygotene spermatocytes; P, pachytene spermatocytes; D, diplotene spermatocytes; P‐like, pachytene‐like spermatocytes; apo, apoptotic cells. Scale bars = 50 µm. B) Testis/body weight curves of control and hnRNPC cKO mice from P7 to P56 (n = 3 per group). Data are presented as mean ± SD, and a two‐tailed Student's *t*‐test was performed. ^*^ *p *<0.05, ^**^ *p *<0.01, ^***^ *p *<0.001. C) Representative confocal images of IF with anti‐γH2AX antibody (green), anti‐SYCP3 antibody (red), and DAPI (blue) on testis sections from P14 to P56 control and hnRNPC cKO mice are shown. Scale bars = 50 µm. D) Representative confocal images of IF with anti‐c‐KIT antibody (green), anti‐DDX4 antibody (red), and DAPI (blue) on testis sections from P8 control and hnRNPC cKO mice are shown. Scale bars = 50 µm. E) Quantification of the c‐KIT^+^ cells per tubule from (D) (n = 3 per group). Data are presented as mean ± SD and a two‐tailed Student's *t*‐test was used. ns, not significant. F) Representative confocal images of IF with anti‐STRA8 antibody (green), anti‐SYCP3 antibody (red), and DAPI (blue) on testis sections from P10 control and hnRNPC cKO mice are shown. Scale bars = 50 µm. G,H) Proportion of tubules with robust STRA8 expression (G) and the quantification of STRA8^+^SYCP3^+^ cells per tubule (H) of control and hnRNPC cKO testes at P10 (n = 3 per group). Data are presented as mean ± SD, and statistical test is a two‐tailed Student's t‐test. ^*^ *p *<0.05, ^**^ *p *<0.01. I) Representative confocal images of IF with anti‐STRA8 antibody (green), EDU (purple), anti‐PLZF antibody (red), and DAPI (blue) on testis sections from P10 control and hnRNPC cKO mice are shown. Scale bars = 50 µm. J) Quantification of the PLZF^−^ STRA8^+^ EDU^+^ cells per tubule from (I) (n = 3 per group). Data are presented as mean ± SD and a two‐tailed Student's *t*‐test was used. ^*^ *p *<0.05.

To determine the cause of sterility in hnRNPC cKO males, we conducted histological analyses and found that pachytene‐like spermatocytes were the most advanced spermatogenic cells in hnRNPC cKO adult testes, and no mature spermatozoa were observed in hnRNPC cKO cauda epididymis (**Figure** **2**A). To assess the time point at which defective spermatogenesis occurs in hnRNPC cKO testes, we collected testes at different developmental stages from P7 to P56 at weekly intervals and found that the testis size of hnRNPC cKO mice became significantly smaller as early as P14 (Figure 2B). Therefore, we evaluated meiotic abnormalities by co‐staining SYCP3 (a marker of meiotic chromosome axis) and γH2AX (a marker of DNA damage response for meiotic recombination and XY body) at different developmental stages of testes (P14, P21, P28, P35, and P56). Normally, γ‐H2AX diffusely distributes in leptotene and zygotene spermatocytes but only decorates the condensed XY body in pachytene and diplotene spermatocytes. In control testes, pachynema or diplonema was present from P14, but in hnRNPC cKO testes it didn't appear until P28, which showed “dot‐like” γH2AX signals (Figure 2C). Co‐staining of SYCP3 with H1T confirmed the absence of mid‐pachynema in P14 hnRNPC cKO testes and the delayed appearance of pachynema in P28 hnRNPC cKO testes (Figure S3A,B, Supporting Information). Further TUNEL (terminal dUTP nick‐end labeling) staining revealed a significant increase in apoptotic cells at all three stages detected (P14, P21, and P56) in hnRNPC cKO testes compared to controls (Figure S3C,D, Supporting Information). These data suggest that ablation of hnRNPC in male germ cells causes delayed meiotic progression in mice.

Given the prominent expression of hnRNPC in spermatogonia, we investigated whether spermatogonia development is affected in hnRNPC cKO mice by IF assays. The results showed that the number of c‐KIT^+^ differentiated spermatogonia was comparable between hnRNPC cKO and control testes at P8 (Figure 2D,E), indicating that spermatogonial differentiation was not affected. Since meiosis is initiated by retinoic acid induction of *Stra8*,^[^ ^34^ ^]^ we then assessed meiotic initiation by examining STRA8. In particular, the reduced number of robust STRA8^+^ tubules and STRA8^+^SYCP3^+^ spermatocytes in P10 hnRNPC cKO testes was observed, indicating that meiotic initiation is disrupted in hnRNPC cKO mice (Figure 2F–H). Furthermore, we performed co‐immunostaining of STRA8 with PLZF at P10 testes and found that preleptotene spermatocytes (labeled STRA8^+^PLZF^−^) were significantly reduced in hnRNPC cKO testes compared to control (Figure S3E,F, Supporting Information), confirming that hnRNPC depletion in the testis impairs meiotic initiation and progression. To further investigate the cause of cell loss, we used cleaved CASPASE3 to label apoptotic cells and found no difference between control and hnRNPC cKO mice (Figure S3G, Supporting Information). Since DNA is replicated just before germ cells enter into meiotic prophase I,^[^ ^35^ ^]^ we injected EDU into P10 control and hnRNPC cKO male mice to determine premeiotic DNA replication. The results showed that PLZF^−^STRA8^+^EDU^+^ preleptotene spermatocytes were reduced in hnRNPC cKO testes, indicating that premeiotic DNA replication was affected upon hnRNPC depletion (Figure 2I,J). Taken together, these results suggest that hnRNPC depletion in the testis impairs meiotic initiation and progression, thereby leading to defective spermatogenesis and male infertility.

### 2.3 hnRNPC‐Deficient Spermatocytes Fail to Progress Through Meiotic Prophase

**Figure 3:**
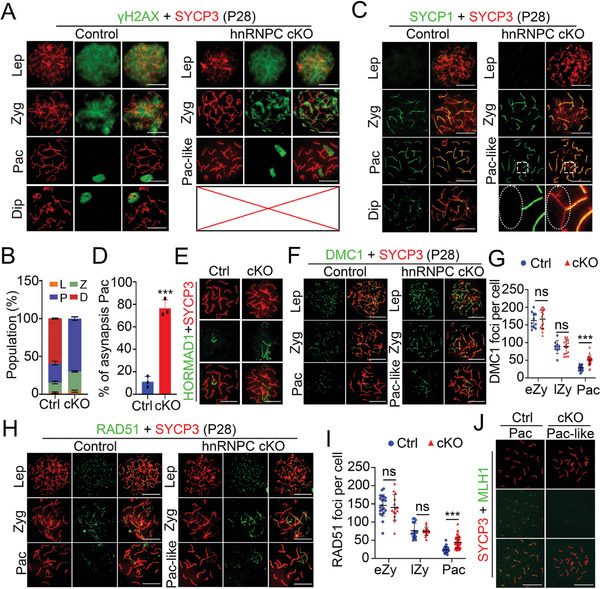
hnRNPC depletion causes defects in synapsis and meiotic recombination. A) Immunostaining with anti‐γH2AX antibody (green) and anti‐SYCP3 antibody (red) on nuclear spreading spermatocytes from P28 control and hnRNPC cKO mouse testes. Lep, leptotene; Zyg, zygotene; Pac, pachytene; Dip, diplotene; Pac‐like, pachytene‐like. Scale bars = 5 µm. B) Histogram showing percentages of population for each type of spermatocyte, including leptotene (L), zygotene (Z), pachytene (P), and diplotene (D) stages. Data are presented as mean ± SD, n = 3 per group. C) Co‐immunostaining of SYCP1 (green) and SYCP3 (red) in nuclear spreading spermatocytes of P28 control and hnRNPC cKO mouse testes. Scale bars = 5 µm. D) Histogram showing the percentage of abnormal synapsis of pachytene spermatocytes in control and hnRNPC cKO testes at P28 (n = 3 per group). Data are presented as mean ± SD, and statistical test is a two‐tailed Student's *t*‐test. ^***^ *p *<0.001. E,F) Representative images of immunostaining with anti‐HORMAD1 antibody (green) (E) or anti‐DMC1 antibody (green) (F) and anti‐SYCP3 antibody (red) on nuclear spreading spermatocytes from P28 control and hnRNPC cKO mouse testes are shown. Lep, leptotene; Zyg, zygotene; Pac, pachytene; Pac‐like, pachytene‐like. Scale bars = 5 µm. G) Quantification of the number of DMC1 foci number in early zygotene (eZy), late zygotene (lZy), and pachytene (Pac) spermatocytes (n = 3 per group). Data are presented as mean ± SD and statistical test is a two‐tailed Student's *t*‐test. ns, not significant, ^***^ *p *<0.001. H) Representative images of IF with anti‐RAD51 antibody (green) and anti‐SYCP3 antibody (red) on nuclear spreading spermatocytes from P28 control and hnRNPC cKO mouse testes are shown. Lep, leptotene; Zyg, zygotene; Pac, pachytene; Pac‐like, pachytene‐like. Scale bars = 5 µm. I) Quantification of the number of RAD51 foci number in early zygotene (eZy), late zygotene (lZy), and pachytene (Pac) spermatocytes (n = 3 per group). Data are presented as mean ± SD and statistical test is a two‐tailed Student's *t*‐test. ns, not significant, ^***^ *p *<0.001. J) Representative images of IF with anti‐MLH1 antibody (green) and anti‐SYCP3 antibody (red) on nuclear spreading spermatocytes from P28 Ctrl and cKO mouse testes are shown. Pac, pachytene; Pac‐like, pachytene‐like. Scale bars = 5 µm.

To further determine how the meiotic process was impaired in hnRNPC cKO mice, we performed chromosome spreading to examine meiotic progression by co‐immunostaining SYCP3 with γH2AX, SYCP1 (a marker of homologous synapsis), and HORMAD1 (a marker known to be localized to the unsynapsed chromosome axis to promote DSB formation and disappear from autosomes upon completion of synapsis^[^ ^36^ ^]^). Co‐staining of γH2AX together with SYCP3 showed that hnRNPC cKO spermatocytes initiated DSBs for meiotic recombination and underwent homologous chromosome synapsis as in the controls; however, spermatocytes later than pachytene were absent in hnRNPC cKO testes, whereas age‐matched control spermatocytes had already passed through meiotic prophase (**Figure** **3**A). Notably, in addition to the XY body, γH2AX still persisted along synapsed autosomes despite the completion of homologous synapsis in hnRNPC cKO pachytene spermatocytes (termed pachytene‐like), suggesting that DSBs repair is defective upon hnRNPC deletion (Figure 3A). In addition, a much higher proportion of zygotene and pachytene‐like spermatocytes but no diplotene spermatocytes were found in hnRNPC cKO testes at P28 compared to controls (Figure 3B), suggesting a spermatogenic arrest at the pachytene stage in the absence of hnRNPC. Subsequent immunostaining of SYCP1 with SYCP3 showed that SYCP1 colocalized with SYCP3 along the axis of homologous chromosomes except for the XY chromosomes in control pachynema; however, SYCP1 was absent on some autosomes in 76.4% of hnRNPC cKO pachytene‐like spermatocytes (Figure 3C,D), indicating defects in synapsis between homologous chromosome pairs upon hnRNPC deletion. Moreover, HORMAD1 signals were aberrantly retained on the autosomes of pachytene‐like spermatocytes in hnRNPC cKO testes (Figure 3E), further demonstrating the asynapsis phenotype in the mutant mice.

Since meiotic recombination is tightly dependent on the successful repair of programmed DSBs^[^ ^37^ ^]^ and hnRNPC cKO pachytene‐like spermagocytes exhibited abnormal DSB retention on the autosome, we next investigated whether meiotic recombination is affected in hnRNPC cKO mice by co‐immunostaining SYCP3 with RAD51 or DMC1 (two markers of meiotic recombination). During normal meiotic progression, the number of DMC1 foci showed a gradual decrease from early to late zygotene spermatocytes to pachytene spermatocytes (Figure 3F,G). Although the number of DMC1 foci showed no obvious change in hnRNPC cKO early and late zygotene spermatocytes, many DMC1 foci remained on the autosomes in hnRNPC cKO pachytene‐like spermatocytes, whereas the majority of DMC1 foci disappeared from the autosomes with the completion of DSB repair in control pachytene spermatocytes (Figure 3F,G). Similar results were reproducibly obtained using RAD51 staining (Figure 3H,I), suggesting severe defects in meiotic recombination caused by hnRNPC deletion. Since DSB repair and subsequent homologous synapsis are required for crossover formation, we then tested whether crossover formation is influenced by hnRNPC deletion by immunostaining for MLH1 (a marker of crossover recombination). As shown in Figure 3J, MLH1 was present in control mid‐late pachynema but could not be detected in hnRNPC cKO pachytene‐like spermatocytes, indicating that hnRNPC ablation causes defective crossover formation in meiosis. Together, these results demonstrate that hnRNPC is required for the completion of the meiotic prophase and that its deletion disrupts synapsis, DSB repair, and crossover formation.

### 2.4 hnRNPC is Required for Oogenesis and Female Fertility in Mice

**Figure 4:**
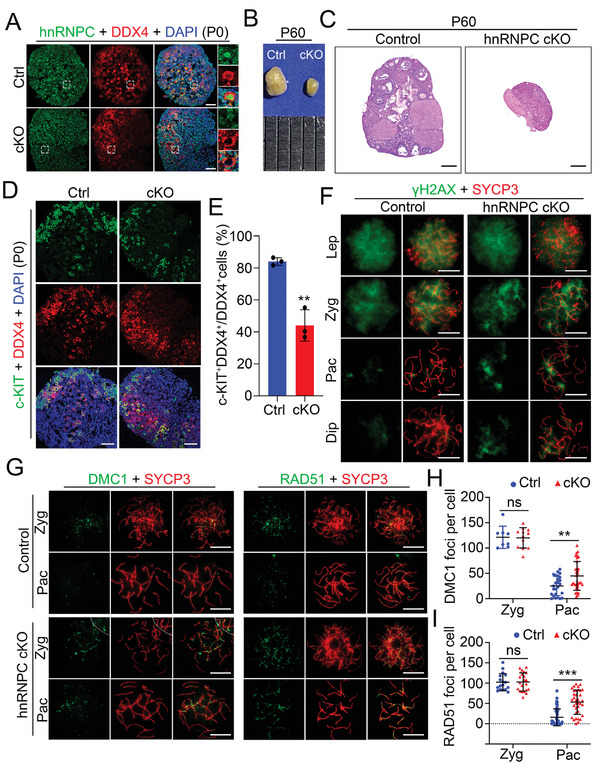
hnRNPC is crucial for female fertility in mice. A) Representative images of IF with anti‐hnRNPC antibody (green), anti‐DDX4 antibody (red), and DAPI (blue) on ovarian sections from control (Ctrl) and hnRNPC cKO (cKO) mice at P10 are shown. Scale bars = 100 µm. B) Gross morphology of ovaries harvested from Ctrl and cKO mice at P60. C) Hematoxylin and eosin (H&E) staining on ovarian sections from control and hnRNPC cKO mice at P60. Scale bars = 100 µm. D) Representative confocal images of IF with anti‐c‐KIT antibody (green), anti‐DDX4 antibody (red), and DAPI (blue) on ovarian sections from Ctrl and cKO mice at P0 are shown. Scale bars = 100 µm. E) Quantification of the ratio of c‐KIT ^+^ DDX4 ^+^cells in DDX4 ^+^ cells of Ctrl and cKO ovaries for (D). Data are presented as mean ± SD, n = 3 per group. A two‐tailed Student's *t*‐test was used for statistical analysis. ^**^ *p *<0.01. F,G) Immunostaining with anti‐γH2AX (F) or anti‐DMC1/RAD51 (G) antibody (green) and anti‐SYCP3 antibody (red) on nuclear spreading oocytes from embryonic day 16.5 (E16.5) control and hnRNPC cKO mice are shown. Lep, leptotene; Zyg, zygotene; Pac, pachytene; Dip, diplotene. Scale bars = 5 µm. H,I) Quantification of the number of DMC1 (H) or RAD51 (I) foci in zygotene (Zyg) and pachytene (Pac) oocytes in (G). Data are presented as mean ± SD, n = 3 biologically independent animals per group. A two‐tailed Student's *t*‐test was used for statistical analysis. ns, not significant, ^**^ *p *<0.01, ^***^ *p *<0.001.

Since *Stra8‐GFP* Cre could also mediate the deletion of target genes in oocytes,^[^ ^38^ ^]^ we investigated the role of hnRNPC in female germ cells. Similar with its expression pattern in males, hnRNPC appeared in pachytene oocytes and exhibited a higher expression in diplotene oocytes (Figure S4A, Supporting Information), suggesting that hnRNPC may function in female meiosis. Further, IF assays revealed that hnRNPC was highly expressed in oocytes derived from P0 control ovaries and was successfully deleted in the hnRNPC cKO oocytes (**Figure** **4**A). Interestingly, we found that the size of hnRNPC cKO ovaries in both P60 and P21 mice was dramatically reduced compared to age‐matched control ovaries (Figure 4B; Figure S4B, Supporting Information), and the adult hnRNPC cKO female mice were also completely infertile. Histological analysis of the ovaries revealed that there were no follicles left in the hnRNPC cKO ovaries at P60 and very few follicles were observed in the hnRNPC cKO ovaries at P21 (Figure 4C; Figure S4C, Supporting Information). In addition, immunohistochemistry for DDX4 showed that the total number of oocytes was consistently reduced in the hnRNPC cKO ovaries at both P1 and P3 (Figure S4D, Supporting Information). Together, these results indicate that hnRNPC is required for oocyte development and female fertility.

To explore the cause of the decrease in oocyte number in hnRNPC cKO mice, we next detected c‐KIT, a marker of primordial follicle formation in newborn female mice,^[^ ^39^ ^]^ by IF assays. The results showed that the number of c‐KIT^+^ germ cells was significantly lower in hnRNPC cKO ovaries than that in controls (Figure 4D,E), suggesting that primordial follicle formation was impaired in the absence of hnRNPC. In addition, TUNEL staining revealed more apoptotic cells in hnRNPC cKO ovaries at P0 compared to controls (Figure S4E, Supporting Information). Notably, we found that γH2AX signals only remained in only a few SYCP3^+^ cells in control ovaries at P0, whereas γH2AX‐positive oocytes were significantly increased in hnRNPC cKO ovaries at P0 (Figure S4F,G, Supporting Information). Consistently, chromosome spreading assays revealed the abnormal accumulation of γH2AX signals on the chromosomes of hnRNPC cKO pachytene and diplotene oocytes, indicating impaired DSB repair in hnRNPC cKO oocytes (Figure 4F). Furthermore, we found a higher number of oocytes with compromised synapsis (abnormal SYCP1 expression) in hnRNPC cKO ovaries (Figure S4H,I, Supporting Information) and increased DMC1 and RAD51 foci on the chromosome axes of hnRNPC cKO pachytene oocytes compared to controls (Figure 4G–I), suggesting that hnRNPC depletion in oocytes could lead to defective synapsis and abnormal homologous recombination in meiosis. Taken together, these data indicate that hnRNPC is also essential for female fertility and meiosis, demonstrating the conserved and essential role of hnRNPC in male and female meiotic progression and germ cell development.

### 2.5 hnRNPC Regulates Alternative Splicing of mRNA in Germ Cells

**Figure 5:**
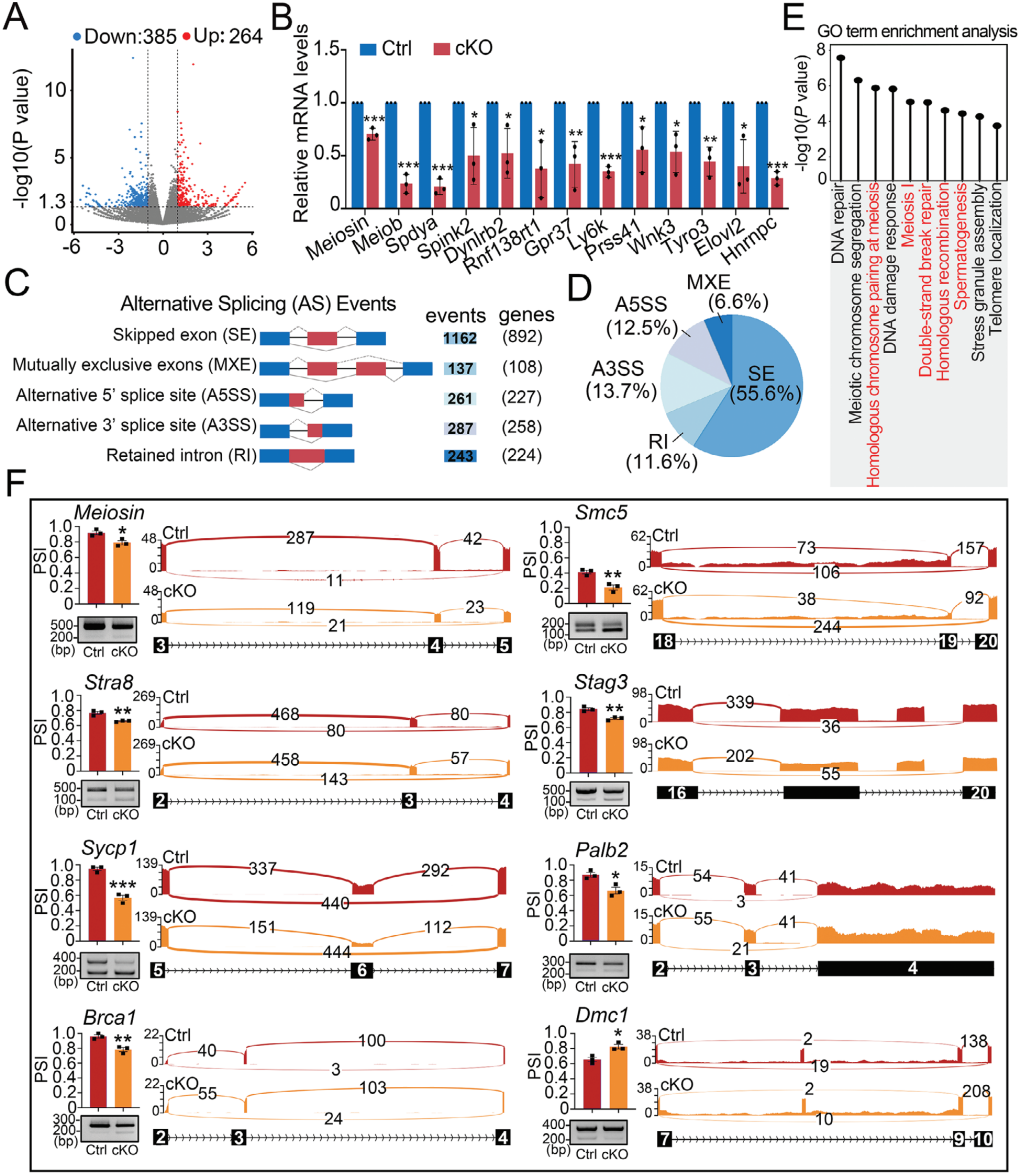
hnRNPC regulates pre‐mRNA alternative splicing in differentiated spermatogonia. A) Volcano map of differentially expressed genes (DEGs) determined by RNA‐seq analysis of differentiated spermatogonia purified from WT and hnRNPC cKO mice at P8. Blue dots indicate downregulated genes and red dots represent upregulated genes. B) Real‐time qPCR analysis of DEGs in differentiated spermatogonia. Data are expressed as mean ± SD of at least three independent experiments. Statistical test is two‐tailed Student's *t*‐test. ^*^ *p *<0.05, ^**^ *p *<0.01, ^***^ *p *<0.001. C) Five alternative splicing (AS) events significantly affected by hnRNPC depletion in the differentiated spermatogonia at P8. The number of predicted AS events and genes in each category upon hnRNPC depletion are indicated. D) Pie charts showing the distribution of regulated splicing events among different splicing types in hnRNPC cKO versus control differentiated spermatogonia. E) Gene ontology (GO) term for genes with the abnormal skipped exon (SE) events in hnRNPC cKO c‐KIT^+^ spermatogonia. F) RT‐PCR analyses for ectopic splicing of meiosis‐related genes (*Meiosin, Stra8, Sycp1, Brac1, Smc5, Stag3, Palb2, Dmc1*) between Ctrl and cKO differentiated spermatogonia. Visualization of the differentially spliced genes are shown using the Integrative Genomics Viewer (IGV). Histograms show the quantification of the percent spliced‐in (PSI) value derived from RNA‐seq data. Data are presented as mean ± SD, n = 3. A two‐tailed Student's *t*‐test was used for statistical analysis. ^*^ *p *<0.05, ^**^ *p *<0.01.

To explore the molecular regulation of hnRNPC in germ cell development, we performed transcriptome analysis on purified c‐KIT^+^ germ cells from control and hnRNPC cKO testes at P8 by magnetic activated cell sorting (MACS). Immunostaining revealed that no fluorescent signal of hnRNPC was observed in hnRNPC cKO c‐KIT^+^ cells (Figure S5A, Supporting Information), suggesting a high Cre efficiency in our *Cre/loxP* system. Since c‐KIT is expressed in Leydig cells,^[^ ^40^ ^]^ we also used c‐KIT and CYP17A1 (a marker for Leydig cells) antibodies to stain the isolated c‐KIT^+^ and interstitial cells and found that the isolated c‐KIT^+^ cells showed almost no CYP17A1 signals, indicating their high purity (Figure S5B, Supporting Information). Further RNA‐seq analysis showed that a total of 385 downregulated genes and 264 upregulated genes (fold‐change > 2 and *p *<0.05) were identified in hnRNPC cKO differentiated spermatogonia (c‐KIT^+^ cells) compared to controls (**Figure** **5**A; Table S1, Supporting Information). Gene ontology (GO) term analysis revealed that these differentially expressed genes (DEGs) are involved in cellular responses to interferon‐beta, cell‐cell adhesion, developmental cell growth, etc. (Figure S5C,D, Supporting Information). Many downregulated genes have been reported to be involved in spermatogenesis, such as *Meiosin*, which encodes a key protein involved in meiosis initiation by interacting with STRA8.^[^ ^2^ ^]^ We then selected 12 downregulated genes related to spermatogenesis, including *Meiosin*, *Meiob*, *Spdya*, *Spink2*, *Dynlrb2*, *Rnf138rt1*, *Gpr37*, *Ly6k*, *Prss41*, *Wnk3*, *Tyro3*, *and Elovl2*, and verified their mRNA levels using RT‐qPCR (Figure 5B).

Since hnRNP proteins were initially discovered as regulators of alternative splicing (AS),^[^ ^41^ ^]^ we next explored whether hnRNPC regulates AS events in differentiated spermatogonia. Based on RNA‐seq data, a total of 2090 abnormal AS events (1445 genes were involved) were found in hnRNPC cKO germ cells, including 1162 skipped exons (SE) (≈55.6%), 287 alternative 3′ splice sites (A3SS) (≈13.7%), 261 alternative 5′ splice sites (A5SS) (≈12.5%), 243 retained introns (RI) (≈11.6%) and 137 mutually exclusive exons (MXE) (≈6.6%) (Figure 5C,D; Table S2, Supporting Information). By comparing the DEGs with 878 genes with abnormal alternative splicing, we found that only 31 genes overlapped (Figure S5E, Supporting Information), suggesting that there are different regulatory mechanisms between DEGs and splicing. Further, GO term enrichment analysis revealed that genes harboring abnormal SE were mainly involved in DSB repair, homologous recombination, and spermatogenesis (Figure 5E). Among the abnormal splicing events, we mainly focused on SE events as this was the most prominent type of AS. Nine genes were selected for verification by RT‐PCR in c‐KIT^+^ germ cells, which showed that loss of hnRNPC resulted in abnormal alternative splicing of *Meiosin, Stra8, Sycp1, Brca1, Smc5, Stag3, Palb2*, *Ankrd31*, and *Dmc1* (Figure 5F; Figure S5F, Supporting Information), consistent with RNA‐seq data. We then detected the protein level of some meiosis‐related genes (e.g., *Stra8*, *Sycp1*, *Dmc1*, and *Palb2*) using P10 germ cells and found that their protein levels decreased upon hnRNPC deletion (Figure S5G,H, Supporting Information). Taken together, these results suggest that hnRNPC ablation may affect meiotic process by regulating the expression of some meiosis‐related genes at the transcriptional and translational levels, thereby controlling male germ cell development.

### 2.6 hnRNPC Functions in an m6A‐Dependent Manner During Germ Cell Development

**Figure 6:**
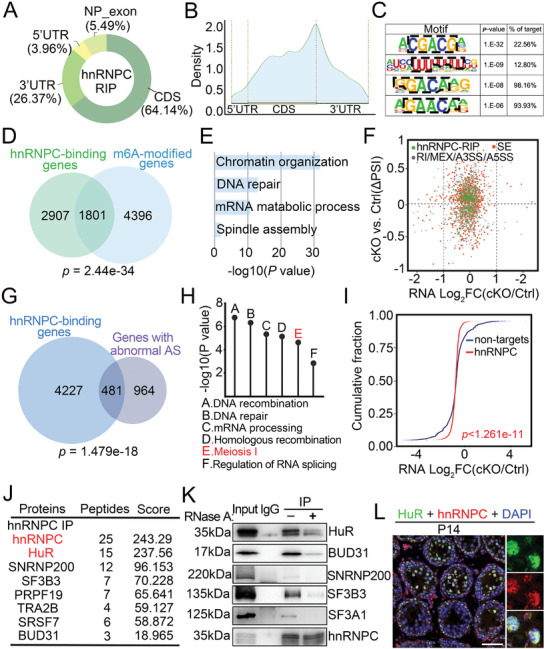
hnRNPC acts as an m6A reader and interacts with HuR. A) Doughnut chart showing the distribution region of hnRNPC‐binding peaks in differentiated spermatogonia of the mouse genome. B) Distribution of m6A‐binding peaks along with transcripts. C) hnRNPC‐binding motifs identified by RIP‐seq in differentiated spermatogonia.D) Venn diagram showing the overlap of hnRNPC‐binding genes and m6A‐modified genes in differentiated spermatogonia. E) GO term analysis of the 1801 overlapping genes in (D).F) Scatter plots of hnRNPC RIP‐seq versus RNA‐seq in Ctrl and cKO spermatogonia. Genes were classified into groups according to the indicated criteria. Green dots indicate genes bound by hnRNPC. Red dots indicate genes with abnormal SE in cKO differentiated spermatogonia, and grey dots indicate genes with other 4 types of abnormal AS in cKO differentiated spermatogonia. G) Venn diagram showing overlap of genes bound by hnRNPC and genes with abnormal AS in cKO differentiated spermatogonia. H) GO term analysis of hnRNPC binding genes with abnormal AS in cKO differentiated spermatogonia. I) Cumulative distribution of RNA abundance changes between Ctrl and cKO differentiated spermatogonia. Non‐targets are marked in blue and hnRNPC targets are shown in red. J) A list of eight candidate hnRNPC‐interacting proteins identified by IP‐MS from P8 mouse testes. K) Immunoprecipitation (IP) with anti‐hnRNPC antibody and control IgG with or without RNase A treatment in P8 WT mouse testes. Immunoprecipitates were blotted with the indicated antibodies. L) Representative confocal images of IF with anti‐HuR antibody (green), anti‐hnRNPC antibody (red), and DAPI (blue) on testis sections from WT mice at P14. Scale bars = 50 µm.

To evaluate whether the function of hnRNPC in spermatogenesis depends on its role as an m6A reader, we conducted hnRNPC RIP‐seq and meRIP‐seq analyses in purified c‐KIT^+^ differentiated spermatogonia. A total of 20917 hnRNPC‐binding peaks from 4709 genes were identified in two biological replicates (Table S3, Supporting Information), most of which were enriched in the CDS (64.14%) and 3′ UTR (26.37%) regions of mRNAs (**Figure** **6**A), which is consistent with characteristic distributions of m6A modification from previously reported^[^ ^42^ ^]^ and our meRIP‐seq data (Figure 6B; Table S4, Supporting Information). Motif analysis revealed a key consensus m6A sequence CGACG^[^ ^43^ ^]^ among the hnRNPC binding motifs (Figure 6C). Consistently, a previously reported hnRNPC binding motif (U‐tracts) and a known m6A consensus motif GRACH^[^ ^24^ ^]^ were also present in our RIP‐seq peak sets (Figure 6C). Further, we overlapped hnRNPC‐binding genes with m6A‐modified genes and found that 1801 out of 4708 (38.3%, *p* = 2.44e‐34) hnRNP‐binding genes carried m6A modifications (Figure 6D). Provokingly, the overlapping genes are mainly related to important pathways in germ cell development, including chromatin organization, DNA repair, and mRNA metabolism (Figure 6E). These results suggest that hnRNPC may function in an m6A‐dependent manner during spermatogenesis and male germ cell development.

We then conducted a combined analysis of the hnRNPC‐binding transcripts and AS‐changed genes in the RNA‐seq data and found that many genes with abnormal alternative splicing were bound by hnRNPC (Figure 6F). In addition, 481 of all 1445 genes (*p* = 4.35e‐12) harboring aberrant AS events overlapped with hnRNPC‐binding genes (Figure 6G) and, importantly, these overlapping genes (e.g., *Sycp1, Brca1, Smc5, Palb2, Ankrd31, Sun1, etc*.) were mainly involved in critical processes of spermatogenesis such as DNA repair, homologous recombination and meiosis I (Figure 6H), further supporting that hnRNPC could regulate the AS of some meiosis‐related genes by directly binding them. We then analyzed the cumulative distribution of RNA abundance between control and cKO spermatogonia and found that some hnRNPC targets were more abundant in the presence of hnRNPC, while others were less abundant (Figure 6I), suggesting that hnRNPC had no bias toward stabilizing or destabilizing targets.

### 2.7 hnRNPC Interacts with HuR to Modulate its Target Genes in Germ Cells

To elucidate the molecular mechanisms by which hnRNPC regulates germ cell development, we first performed immunoprecipitations using antibody against hnRNPC followed by mass spectrometry analysis (IP‐MS) and identified a total of 260 candidate hnRNPC‐interacting proteins, particularly involved in mRNA splicing, as revealed by GO and KEGG enrichment analysis (Figure S5I,J, Table S5, Supporting Information). We then performed co‐immunoprecipitation (co‐IP) using P8 testes and verified the interaction between hnRNPC and five splicing factors with high scores, including HuR, BUD31, SNRNP200, SF3B3, and SF3A1 (Figure 6J,K). Interestingly, the interactions between hnRNPC and its five interacting partners were remarkably crippled after ribonuclease A (RNase A) treatment, implying that their interactions are partially dependent on the presence of RNA (Figure 6K). Given that HuR has been reported to be required for meiotic progression and that its depletion in spermatogenic cells causes male sterility,^[^ ^32^ ^]^ as well as its strong interaction with hnRNPC, we focused most of our subsequent studies on the relationship between hnRNPC and HuR.

**Figure 7:**
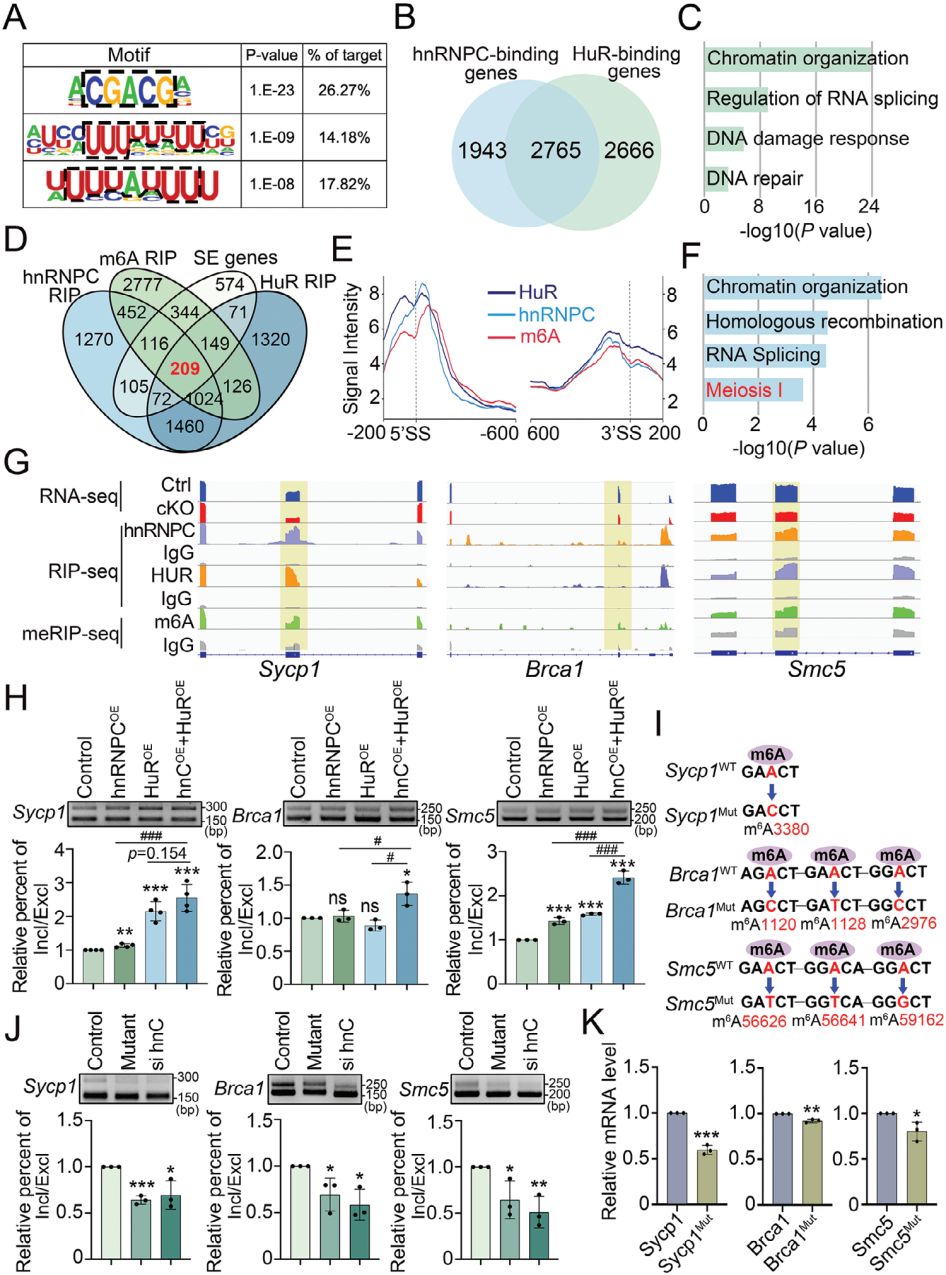
hnRNPC cooperates with HuR to regulate alternative splicing. A) HuR binding motifs identified by RIP‐seq in differentiated spermatogonia. B,C) Venn diagram (B) and GO term analysis (C) of 2765 genes bound by both hnRNPC and HuR in differentiated spermatogonia. D) Venn diagram showing 209 overlapping genes of m6A modified genes (m6A Rip), genes with abnormal SE (SE genes), hnRNPC binding genes, and HuR binding genes. E) Distribution of the 209 overlapping genes near splice sites (200 nt upstream and 600 nt downstream of 5′ splice sites, 5′ SS; 600 nt upstream and 200 nt downstream of 3′ splice sites, 3′ SS). F) GO term analysis of the 209 overlapping genes identified in (D). G) IGV tracks of *Sycp1*, *Brca1*, and *Smc5* in RNA‐seq, hnRNPC RIP‐seq (purple), HuR RIP‐seq (yellow), and meRIP‐seq (green). H) Minigene splicing assay verifying exon skipping of the three selected genes, including *Sycp1*, *Brca1*, and *Smc5* construct. Minigenes were co‐transfected with plasmids expressing Flag‐hnRNPC and/or Myc‐HuR into HEK293 cell line, then their splicing changes were detected by RT‐PCR (top) and statistical analysis (bottom). hnRNPC^OE^ or hnC^OE^, hnRNPC overexpression; HuR^OE^, HuR overexpression. Incl/Excl indicates the percentage of inclusion (upper band) and exclusion (lower band) within exon 6 of *Sycp1*, exon 3 of *Brca1*, and exon 19 of *Smc5* transcript, respectively. Data are expressed as mean ± SD of at least three independent experiments. A two‐tailed Student's *t*‐test was used for statistical analysis. ^*^indicate comparison between Ctrl and other groups. ns, not significant, ^*^ *p*, ^#^ *p *<0.05, ^**^ *p *<0.01, ^***^ *p*, ^###^ *p *<0.001. I) Schematic of mutant construction of the three minigenes (*Sycp1*, *Brca1*, and *Smc5*) at the predicted m6A modification sites. J) Minigene splicing assay analyzing the level of exon skipping of *Sycp1*, *Brca1*, and *Smc5* constructs. They and their mutants were transfected into HEK293 cell line under the indicated conditions, then their splicing changes were detected by RT‐PCR (top) and statistical analysis (bottom). siHnC, si*Hnrnpc*. Incl/Excl indicates the percentage of inclusion (upper band) and exclusion (lower band) within exon 6 of *Sycp1*, exon 3 of *Brca1*, and exon 19 of *Smc5* transcripts, respectively. Data are expressed as mean ± SD of at least three independent experiments. A two‐tailed Student's *t*‐test was used for statistical analysis. ^*^ *p *<0.05, ^**^ *p *<0.01, ^***^ *p *<0.001. K) hnRNPC RIP‐qPCR showing the binding ability of hnRNPC with the three minigenes (*Sycp1, Brca1*, and *Smc5*) and their mutants (*Sycp1^Mut^, Brca1 ^Mut^* and *Smc5 ^Mut^*). Data are expressed as mean ± SD of at least three independent experiments. A two‐tailed Student's *t*‐test was used for statistical analysis. ^*^ *p *<0.05, ^**^ *p *<0.01, ^***^ *p *<0.001.

We then performed IF analysis to examine the expression pattern of HuR in male germ cells and found that HuR had a similar localization to hnRNPC, with high expression in spermatogonia, pachynema, diplonema, and round spermatids (Figure 6L; Figure S6A–C, Supporting Information). Consistent with the expression pattern of hnRNPC, HuR was also highly expressed in c‐KIT^+^ spermatogonia (Figure S6D, Supporting Information), whereas its subcellular localization was not affected by hnRNPC deletion (Figure S6E, Supporting Information). Furthermore, we found that HuR protein levels were comparable between control and hnRNPC cKO isolated germ cells (Figure S6F, Supporting Information). Given the similar characteristics and expression pattern between hnRNPC and HuR, we next carried out HuR RIP‐seq using purified c‐KIT^+^ spermatogonia and identified a total of 10258 HuR binding peaks from 5431 genes (Table S6, Supporting Information). As expected, HuR binding peaks were predominantly enriched at CDS and 3′ UTR regions (Figure S6G, Supporting Information), similar to the distribution pattern of hnRNPC binding sites and m6A modification sites. Interestingly, we found that the highest ranked binding motif of HuR was the “CGACG”, which is the same as the hnRNPC binding motif (**Figure** **7**A). In addition, two known HuR motifs identified in vitro,^[^ ^44^ ^]^ including “UUUUUUU” and “UUUAUUU”, were also present among our HuR binding peaks, indicating the reliability of the RIP‐seq data. Importantly, we identified 2765 genes were shared between the hnRNPC‐binding (4708) and HuR‐binding (5431) genes that are involved in several key processes in meiosis, including chromatin organization, regulation of RNA splicing, DNA damage response, and DNA repair (Figure 7B,C). We further performed hnRNPC and HuR RIP‐qPCR analyses on selected eight overlapping genes (*Brca1, Slx4, Ankrd31, Sycp1, Bptf, Chfr, Celf1*, and *Traf3ipi*) and found they are all indeed bound by hnRNPC and HuR (Figure S7A–C, Supporting Information). Taken together, these findings suggest that hnRNPC and HuR interact with each other and share many target genes in germ cells, suggesting their functional communication during spermatogenesis.

### 2.8 hnRNPC Cooperates with HuR to Regulate Alternative Splicing in Germ Cells

To further investigate the underlying mechanism of dysregulated AS upon hnRNPC ablation in germ cells, we conducted multi‐omics analyses using our four datasets, including genes with aberrant SE in hnRNPC cKO spermatogonia, genes with m6A modifications, hnRNPC‐binding genes and HuR‐binding genes, and found that 209 genes overlapped among them (Figure 7D). We next assessed the distribution of hnRNPC and HuR binding regions on the 209 target genes and observed that both hnRNPC and HuR showed high affinity near the exon splice sites, including 5′SS and 3′SS, where m6A modifications were also enriched (Figure 7E). GO analysis revealed that the 209 genes were involved in key pathways for spermatogenesis, such as homologous recombination and meiosis I (Figure 7F). We then selected three key meiosis‐related genes (*Sycp1*, *Brca1*, and *Smc5*) for further visualization of the diverse sequencing analyses using the Integrative Genomics Viewer (IGV) and confirmed that the strong binding cites of hnRNPC and HuR are in the regions near their splice sites of three genes, consistent with the distribution pattern with m6A modifications (Figure 7G). To examine the functional interplay between hnRNPC and HuR in alternative splicing, we constructed minigenes for *Sycp1*, *Brca1*, and *Smc5*, which harbor their own alternatively spliced exon, and co‐transfected them with plasmids expressing hnRNPC and/or HuR into the HEK293T cell line. Ectopic overexpression of Flag‐tagged hnRNPC and Myc‐tagged HuR was confirmed by Western blot (Figure S7D, Supporting Information). Interestingly, compared to single hnRNPC or HuR transfection, a higher proportion of exon inclusion (Exon Inclusion Isoform / Exon Exclusion Isoform)was detected in all three detected genes when hnRNPC and HuR were co‐overexpressed (Figure 7H), suggesting functional cooperation between hnRNPC and HuR in regulating alternative splicing.

To determine whether hnRNPC‐mediated regulation of alternative splicing depends on m6A modification, we predicted the m6A sites of the three selected genes (*Sycp1*, *Brca1*, and *Smc5*) using SRAMP (a Sequence‐based RNA Adenosine Methylation site Predictor)^[^ ^45^ ^]^ and mutated their m6A sites with high confidence (Figure 7I). Meanwhile, we knocked down the *Hnrnpc* gene in HEK293T cells by using two independent siRNAs (Figure S7E, Supporting Information) and found that both the m6A site mutation and *Hnrnpc* knockdown can repress exon inclusion events of the three detected genes (Figure 7J), suggesting that hnRNPC regulates their alternative splicing in an m6A‐dependent manner, at least in part. Furthermore, hnRNPC RIP‐qPCR confirmed that its binding affinity to the three target transcripts (*Sycp1*, *Brca1*, and *Smc5*) was significantly reduced when the corresponding m6A sites were mutated (Figure 7K). Together, these data suggest that hnRNPC may regulate alternative splicing through the recognition of m6A sites.

## 3 Discussion

The current study highlights the importance of the potential m6A reader hnRNPC in alternative splicing during spermatogenesis. Within spermatogenic cells, hnRNPC was highly expressed in spermatogonia, pachynema, diplonema, and round spermatids. Germ cell‐specific deletion of hnRNPC resulted in male sterility due to defects in meiotic initiation and progression. In addition, hnRNPC cKO spermatocytes are ultimately arrested at the pachytene stage due to persistent DNA damage, aberrant synapsis, and abnormal DSB repair. Since the *Stra8*‐*GFP* *C* *re* can also induce the deletion of target genes in female germ cells at an embryonic stage when meiosis initiation occurs, we also observed that the hnRNPC cKO females exhibited an infertile phenotype with phenocopied males, suggesting a conserved role of hnRNPC in both male and female gametogenesis.

Meiosis initiation is a vital step in spermatogenesis that is mediated by some key factors, such as STRA8^[^ ^34^ ^]^ and MEIOSIN.^[^ ^2^ ^]^ In this study, the mRNA level of *Meiosin* was significantly decreased and alternative splicing of *Meiosin* and *Stra8* was also altered in hnRNPC cKO mice, which is likely to be the main cause of abnormal meiotic initiation. Moreover, our study showed that hnRNPC depletion can also affect alternative splicing of a large number of genes involved in DSB repair and homologous recombination, such as *Dmc1*, *Brca1, Ankrd31, Palb2*, *Sycp1*, and *Smc5*. Previous studies have reported that BRCA1 and PALB2 form a complex to promote homologous recombination,^[^ ^46^, ^47^ ^]^ and PALB2 is essential for the formation of the BRCA1‐BRCA2‐RAD51 axis, which facilitates homologous recombination repair.^[^ ^48^, ^49^ ^]^ ANKRD31 may interact with REC114, a DSB promoting factor, to promote DSB formation and recombination.^[^ ^50^ ^]^ Therefore, together with these published studies, the abnormal splicing events induced by hnRNPC deletion observed in this study may explain the abnormal meiosis initiation and impaired synapsis and homologous recombination phenotype in hnRNPC cKO spermatocytes. Furthermore, in the current study, some differentially expressed genes (such as *Meiob*, Spdya, and *Spink2*) identified from RNA‐seq data that have been reported to be essential for spermatogenesis. For example, *Meiob* is required for meiotic recombination, and *Meiob*‐null mutants exhibit meiotic failure and sterility in both sexes.^[^ ^51^ ^]^ SPDYA forms a complex with SUN1 and is required for efficient homologous pairing and synapsis, and disruption of their interaction results in male infertility.^[^ ^52^ ^]^ *Spink2* is also critical for spermatogenesis and is involved in serine protease‐mediated apoptosis in male germ cells.^[^ ^53^ ^]^ Although it is unclear how hnRNPC affects the expression of these genes, it cannot be ruled out that these abnormally expressed genes contribute to the defects in spermatogenesis in hnRNPC cKO mice, particularly in meiosis. Interestingly, our study showed a relatively low overlap between DEGs and genes with alternative splicing, probably indicating that the abnormal alternative splicing would not lead to the dysregulation of mRNA expression in hnRNPC cKO germ cells and that hnRNPC may regulate alternative splicing and transcription by other potential unknown mechanisms. Notably, this phenomenon is also observed in previous studies that have identified many splicing factors in germ cell development, including CWF19L2,^[^ ^54^ ^]^ BUD31,^[^ ^55^ ^]^ SRSF1,^[^ ^56^ ^]^ PTBP1,^[^ ^57^ ^]^ etc. In fact, alternative splicing may not change transcription levels, but it can produce mRNAs that differ in their untranslated regions (UTRs) or coding sequence, which could affect mRNA localization or translation.^[^ ^58^ ^]^ Our results showed that the mRNA levels of genes with abnormal splicing were not affected, but the expression levels of target proteins were altered; however, the detailed mechanism needs to be further investigated in the future.

In addition, HuR was demonstrated to be an interplayer of hnRNPC in our current study, and in addition to the similar expression pattern of hnRNPC and HuR in spermatogenic cells, they also share the same binding motif and many target genes, such as *Sycp1, Brca1*, and *Smc5*. Interestingly, the *Vasa*‐Cre‐mediated conditional knockout mouse model showed that targeted deletion of HuR specifically in germ cells results in male but not female sterility,^[^ ^32^ ^]^ in contrast to our *Stra8*‐*GFPCre*‐induced hnRNPC cKO mice, which are infertile in both males and females. The different phenotype in HuR cKO (*Vasa*‐Cre induced) and hnRNPC cKO (*Stra8*‐GFPCre mediated) females could be due to the time of CRE expression in the female germ line, which is earlier at embryonic day 12.5 (E12.5) for *Stra8*‐GFPCre than at E15.5 for *Vasa*‐Cre.^[^ ^59^, ^60^, ^61^ ^]^ Of note, HuR can improve the ability of hnRNPC to promote exon inclusion of the three target genes in vitro, and hnRNPC can also enhance the function of HuR in turn, indicating that they may cooperate to modulate RNA splicing. Indeed, we observed that 209 m6A‐modified genes were bound by both hnRNPC and HuR in regions close to their splicing sites. In addition, recent studies have reported that m6A readers, such as YTHDC1^[^ ^62^ ^]^ and hnRNPG,^[^ ^63^ ^]^ can regulate pre‐mRNA splicing in vitro. Consistently, in this study, we identified ≈45% of genes with abnormal alternative splicing events in hnRNPC mutants showed m6A modifications, suggesting that hnRNPC regulates splicing in an m6A‐modification dependent manner as well. It has been shown that m6A modification of a 3ʹ splice site can inhibit pre‐mRNA splicing in mammals.^[^ ^64^ ^]^ Interestingly, we validated that mutations of m6A modification sites near splicing sites can inhibit exon inclusion of hnRNPC target genes, similar to the effects of *Hnrnpc* knockdown, and severely abrogate the ability of hnRNPC to bind its target genes, highlighting the important role of hnRNPC as an m6A reader in alternative splicing. Previous studies have reported that m6A readers can also regulate mRNA translation or degradation through direct binding of potential m6A sites;^[^ ^65^, ^66^, ^67^ ^]^ therefore, in addition to alternative splicing, we cannot rule out the possibility that hnRNPC may play a critical role in other aspects of RNA metabolism during spermatogenesis.

Notably, many splicing‐changed genes were not directly bound by hnRNPC, but we noticed that some genes with abnormal AS bound directly by hnRNPC can regulate alternative splicing, such as *Celf1*,^[^ ^68^ ^]^ *Hnrnph1*,^[^ ^69^ ^]^ *Srsf11*,^[^ ^70^ ^]^ etc., raising the possibility that hnRNPC could enhance its influence in the regulation of RNA splicing by coordinating splicing factors. Indeed, although a large number of splicing changed genes were identified from our RNA‐seq dataset, many of them were indeed not directly bound by hnRNPC and its partner HuR. Among the hnRNPC interacting candidates, there are also many splicing factors, such as SRSF family members (SRSF3, SRSF7, SRSF10, etc), SNRNP200, SF3B and SF3A family. It therefore remains to be further investigated whether and how hnRNPC regulates RNA splicing in an indirect manner via these splicing factors.

**Figure 8:**
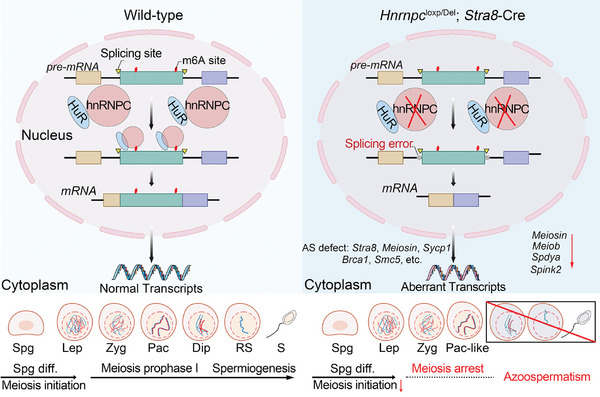
Working model of the role of hnRNPC in spermatogenesis. hnRNPC cooperates with HuR to regulate alternative splicing in an m6A‐dependent manner in male germ cells. Loss of hnRNPC affects meiosis initiation and meiotic progression due to alternative splicing defects of essential spermatogenesis‐related genes.

In conclusion, this study demonstrates that hnRNPC plays a critical role in post‐transcriptional regulation by modulating alternative splicing in germ cells. In male germ cells, hnRNPC may function with HuR to regulate alternative splicing of spermatogenesis‐related genes in an m6A‐dependent manner (**Figure** **8**), thereby controlling meiotic initiation and progression. Thus, these findings provide a new insight into how the m6A modification and its reader protein, hnRNPC, regulate mRNA splicing during germ cell development.

## 4 Experimental Section

### Mice

*Hnrnpc* ^+/lox^ mice were purchased from *GemPharmatech Company*, and *Stra8‐GFPCre* mice were provided by Prof. Minghan Tong of the Chinese Academy of Sciences. hnRNPC cKO (*Stra8‐GFPCre*; *Hnrnpc* ^flox/△^) mice were generated by crossing *Hnrnpc* ^flox/flox^ with *Stra8‐GFPCre*; *Hnrnpc* ^+/flox^. Genotyping was performed by PCR of mouse tail genomic DNA. PCR primer sequences are shown in Table S7 (Supporting Information). All mice used in this study were of C57BL/6J background and were maintained at 22–24 °C with food and water available ad libitum in accordance with the Guide for the Care and Use of Laboratory Animals. All animal procedures were approved by the Institutional Animal Care and Use Committee of Tongji Medical College, Huazhong University of Science and Technology.

### Histological Analysis and Immunostaining

Mouse testes and ovaries were collected and fixed in Bouin's solution (Sigma, HT10132) overnight at room temperature (RT). After dehydration and paraffin embedding, the samples were sectioned at 5 µm thickness. Sections were deparaffinized and rehydrated for hematoxylin and eosin (H&E) staining, periodic acid‐Schiff (PAS) staining, and immunohistochemistry according to a standard protocol. For immunofluorescence, testes were isolated and fixed in 4% paraformaldehyde (PFA) (Sigma, P6148) overnight at 4 °C. After dehydration, frozen sections were cut at 5 µm thickness and boiled in sodium citrate buffer (pH = 6.0) for antigen retrieval. After cooling down to RT, slides were blocked with donkey serum for 1h at RT. Sections were then incubated with primary antibodies at 4 °C overnight. After rinsing with PBS, the sections were incubated with secondary antibodies and DAPI. Images were captured using a Zeiss LSM900 confocal microscope equipped with Zen software. All antibodies used in this study are listed in Table S8 (Supporting Information).

### TUNEL Staining Assay

For TUNEL staining, assays were performed using One Step TUNEL Apoptosis Assay KIT (Meilunbio, MA0223) following the manufacturer's instructions.

### Plasmids, Minigenes, and Cell Transfection

Full‐length of mouse *Hnrnpc* and *Hur* cDNA was cloned into a pCMV vector containing the N‐terminal Flag or c‐Myc epitope Tag. For minigene construction, the *Sycp1*, *Brca1*, and *Smc5* minigenes were amplified from adult mouse testis genomic DNA using the primers listed in Table S7 (Supporting Information). For the m6A mutant minigenes, potential m6A sites were predicted using SRAMP (Sequence‐based RNA Adenosine Methylation site Predictor) and m6A sites with high confidence were selected and mutated (*Sycp1* ^Mut^: A3380C; *Brca1* ^Mut^: A1120C, A1128T, A2976C; *Smc5* ^Mut^: A56626T, A56641T, A59162G). The minigenes and mutants were then cloned into the pCDNA3.1(−) vector and validated by sequencing. HEK293T cells were cultured in the DMEM medium supplemented with 10% fetal bovine serum (Gibco, 10 270 106). Lipo8000 Transfection Reagent (Beyotime) was used for transfection according to the manufacturer's protocol. Cells were harvested after 48 h of transfection, followed by the subsequent analysis.

### RT‐PCR and qPCR

Total RNA was extracted using an RNA Extraction KIT (ZYMO Research, R2050) according to the manufacturer's instructions. Residual genomic DNA was then removed using RNase‐free DNase (Roche, 69182), and 1µg of total RNA was reverse transcribed into cDNAs using the Hiscript® II 1st Strand cDNA Synthesis KIT (R211, Vazyme, Nanjing, China) according to the manufacturer's protocol. qPCR was performed using Cham Q SYBR qPCR Master Mix (Q711‐02, Vazyme, Nanjing, China) on a Quant Studio 3 system (Thermo Fisher). Primers for the qPCR assay are listed in Table S7 (Supporting Information).

### Chromosome Spreading Immunofluorescence Analysis

Chromosome spreading immunofluorescence was performed as described previously.^[^ ^71^ ^]^ Briefly, seminiferous tubules were incubated in hypotonic solution (30 mM Tris, 50 mM sucrose, 17 mM trisodium citrate dihydrate, 5 mM EDTA, 0.5 mM DTT, and 0.5 mM phenylmethylsulphonyl fluoride (PMSF), pH = 8.2) for 1 h and then transferred to a 100 mM sucrose solution. The tubules were minced and suspended in 100 mM sucrose to disperse to single cells. The suspension was dropped onto adhesion microscope slides containing 1% PFA and 0.15% Triton X‐100 for 2 h at RT. The slides were then air dried for ≈2 h at RT and washed with 0.4% Photo‐Flo (Kodak, 1464 510). Finally, the slides were air dried again and stored at −40 °C or used for immunostaining.

### Western Blotting

Testes or cells were rinsed with PBS and lysed in cold lysis buffer (P0013, Beyotime, China) supplemented with phosphatase inhibitor and protease inhibitor cocktail tablets. Proteins were separated by 10% SDS‐PAGE, transferred to PVDF membranes, blocked with 5% non‐fat milk in Tris‐buffered Saline with Tween 20 (TBST, pH 7.4) for 1h at RT, and incubated with the relevant primary antibodies overnight at 4 °C. After washing with TBST, the membranes were incubated with secondary antibodies. Signals were detected using an ECL Chemiluminescence Kit (WBKLS0500, EMD Millipore, USA). All antibodies used are listed in Table S8 (Supporting Information).

### Immunoprecipitation (IP)

P8 testes were homogenized in cell lysis buffer (P0013, Beyotime, China) containing protease inhibitor cocktail and DTT. After centrifugation, the lysates were treated with or without RNase A (1µg mL^−1^) for 30 min. Protein G magnetic beads conjugated with antibodies against hnRNPC or rabbit IgG were then added to the tissue lysates and incubated overnight at 4 °C. After washing the beads with lysis buffer, the samples were boiled in loading buffer at 100 °C for 10 min for Western blotting or mass spectrometry analysis.

### Mass Spectrometry (MS)

Immunoprecipitated proteins from the IgG and hnRNPC groups were separated by SDS‐polyacrylamide gel electrophoresis (PAGE) and stained with SimplyBlue (Thermo Fisher). Following overnight washing, in‐gel digestion was performed. The gel containing the proteins was excised, cut into ≈1mm pieces, and reduced with DTT. Gel pieces were then digested with trypsin overnight after washing and dehydration. Peptides were separated on an EASY‐nLC 1200 UPLC system (Thermo Scientific). The buffers were 0.1% formic acid in water for liquid A and 0.1% formic acid, acetonitrile, and water for liquid B (80% acetonitrile). The gradient of the liquid separation was programmed as follows: 0–2 min, from 2% to 5% B; 2–44 min, from 5% to 28% B; 44–51 min, from 28% to 40% B; 51–53 min, from 40% to 100% B; 53–60 min, hold at 100% B. The separated peptides were analyzed on a Q Exactive mass spectrometer (Thermo Fisher) for 60 min. The detection mode used an m/z window of 350 to 1800 with a resolution of 60 000. The MS/MS data files were processed for protein identification specifically for Mus musculus using MaxQuant 2.0.1.0.

### Isolation of c‐KIT^+^ Cells

Testes were collected from P8 mice and digested with collagenase IV, followed by washing with DMEM and centrifugation at 400 × g for 5 min. The seminiferous tubules were then digested again with trypsin containing DNase I for 3 min, and the single cell suspension was passed through a 40‐µm pore size cell strainer. The suspension was then plated onto 0.1% gelatin‐coated plates and incubated overnight as previously reported.^[^ ^72^ ^]^ After overnight incubation, somatic cells adhered to the culture plates and non‐attached germ cells were collected to isolate c‐KIT^+^ cells. CD117 microbeads (130‐097‐146, Miltenyi Biotec, Germany) were then added to the suspensions and incubated for 20 min at 4 °C in the dark according to the manufacturer's instructions. The beads were washed and the cells were resuspended. A column was then rinsed in the magnetic field of a suitable MACS separator and cell suspensions were added. After three washes with BSA buffer (130‐091‐376, Miltenyi Biotec, Germany), the column was removed from the MACS separator, the appropriate amount of buffer was pipetted onto the column, and the cells that passed through, including c‐KIT^+^ cells, were collected.

### RNA‐Seq and Bioinformatic Analyses

Total RNA was extracted from c‐KIT^+^ cells of WT and *Hnrnpc* cKO mice at P8 using Direct‐zol RNA KIT (R2050, ZYMO Research, USA) according to the manufacturer's instructions. Strand‐specific libraries were generated using the Clontech SMARTer cDNA KIT (Clontech Laboratories, CA, USA, Catalog# 634938) and adaptors were removed by digestion with RsaI. Libraries were sequenced on the DNBSEQ‐T7 platform, with three biological replicates for control and hnRNPC cKO mice. Raw data was generated using PE150 sequencing mode. Clean reads were then mapped to the mouse genome (mm10). Differentially expressed genes in pairwise comparisons were measured using DESeq2 and differential variable splicing events were analyzed using rMATS (v4.1.1) software. Differentially expressed genes were defined using the set parameters (*p *<0.05, fold change > 2). To detect valid alternative splicing events, false discovery rates (FDR) <0.05 and |△PSI| >10% were categorized as differential alternative splicing events.

### RNA Immunoprecipitation Sequencing (RIP‐Seq)

RNA immunoprecipitation (RIP) was conducted using MACS‐purified spermatogonia (c‐KIT^+^ cells) from one hundred P8 WT mice. Cells were UV cross‐linked and transferred to lysis buffer containing 100 mm KCl, 10 mm HEPES (pH = 7.0), 0.5% Triton X‐100, 5 mm MgCl_2_, 1 mm DTT, 0.5% NP‐40, RNase inhibitor (100 U mL^−1^) (Invitrogen) and EDTA‐free proteinase inhibitor (Roche) for lysis and sonication. The 10% lysis sample was stored for “input” and the remainder was used in immunoprecipitation reactions with antibodies. Protein G‐Dyna beads were pre‐incubated with hnRNPC or HuR antibody (or IgG for controls) at RT for 1 h, and the cell lysates were added into it for overnight incubation at 4 °C. The next day, the beads were washed three times with 1 mL of high salt wash buffer (50 m Tris‐HCl pH 7.4, 1 M NaCl, 1% Igepal CA‐630, 1 mM EDTA, 0.1% SDS and 0.5% sodium deoxycholate), followed by a further three wash cycles with 1 mL of wash buffer (20 mM Tris‐HCl pH 7.4, 10 mM MgCl_2_, 0.2% Tween‐20 and 5 mM NaCl) at 4 °C, followed by RNA extraction using RNA Clean & Concentrator (ZYMO Research, R1015). The purity and integrity of the eluted RNA was assessed using an Agilent Bioanalyzer. 1–2 µg of cDNA was synthesized from the extracted RNA using the Clontech SMARTer cDNA KIT (Clontech Laboratories, CA, USA, Catalog# 634938) and adaptors were removed by digestion with RsaI. Two biological replicates were analyzed for each sample. Libraries were constructed and loaded onto the Novaseq 6000 platform. RIP peaks were identified using the R package RIPSeeker (v1.26.0) and annotated using HOMER.

### meRIP‐Seq

Total RNA was extracted from c‐KIT^+^ cells using TRIzol reagent (15596026, Invitrogen, USA). After RNA extraction, DNA digestion was performed using DNaseI. RNA was then quantified by Qubit3.0 using the Qubit^TM^ RNA Broad Range Assay Kit (Q10210, Life Technologies, USA). Polyadenylated RNA enrichment was conducted using 50 µg of total RNA with VAHTS mRNA Capture Beads (N401‐01/02, Vazyme, China), and RNA was fragmented into 100–200 nt. Then 10% of the RNA fragments were stored as “Input” and the rest was used for m6A immunoprecipitation (IP). The specific anti‐m6A antibody (202203, Synaptic Systems, Germany) was applied for m6A immunoprecipitation. RNA samples of both input and IP were prepared using TRIzol reagent. Two biological replicates were analyzed for each sample. Library products corresponding to 200–500 bp were enriched, quantified and finally sequenced on a Novaseq 6000 sequencer using the PE150 model. The m6A peaks were annotated using bedtools (version 2.25.0). Sequence motifs enriched in m6A peak regions were identified using Homer (version 4.10).

### Gene Ontology (GO) Analysis

GO analysis was performed using Metascape (http://metascape.org) as described in the previous publication.^[^ ^73^ ^]^ The representation factor calculation was performed to determine whether the expected number of overlaps was found, using the Lund lab's online resource available at http://nemates.org/MA/progs/overlap_stats.html. The exact hypergeometric distribution or the normal approximation of the hypergeometric distribution was used to calculate the significance of overlap.

### Statistical Analyses

The data were shown as the mean ± standard deviation (SD). GraphPad Prism 9.0 software (GraphPad, San Diego, CA, USA) was used for the statistical analyses. Statistical analysis was conducted by one‐way ANOVA or the student's *t*‐test. When *p*<0.05, the difference between groups was considered significant. *p* values are denoted in figures or figure legends by ^*^ *p* or ^#^ *p *<0.05, ^**^ *p *<0.01, and ^***^ *p* or ^###^ *p *<0.001, ns, not significant.

## Conflict of Interest

The authors declare no conflict of interest.

## Author Contributions

X.X., S.F., and X.M. contributed equally to this work. X.X., S.F., and S.Y. conceived and designed the study. X.X., S.F., X.M., K.L., Y.G., B.C., X.F., F.W., and X.W. performed the bench experiments and data analyses. X.M. and Y.G. performed the bioinformatic analysis. X.X. wrote the manuscript. S.Y. and X.W. revised the manuscript. S.Y. supervised the project. All authors read and approved the manuscript.

## Supporting information



Supporting Information

Supplemental Table S1

Supplemental Table S2

Supplemental Table S3

Supplemental Table S4

Supplemental Table S5

Supplemental Table S6

Supplemental Table S7

Supplemental Table S8

## Data Availability

The data that support the findings of this study are available from the corresponding author upon reasonable request.
