# Supplementary material for: hnRNPC Functions with HuR to Regulate Alternative Splicing in an m6A‐Dependent Manner and is Essential for Meiosis
Source: Adv Sci (Weinh). 2025 Feb 8;12(13):2412196. doi: 10.1002/advs.202412196 (PMC11967818; doi:10.1002/advs.202412196)
Supplement: Supplementary file 1 — Supporting Information [file ADVS-12-2412196-s005.pdf]

ADVANCED SCIENCE
Open Access

Supporting Information

for *Adv. Sci.*, DOI 10.1002/advs.202412196

hnRNPC Functions with HuR to Regulate Alternative Splicing in an m6A-Dependent Manner and is Essential for Meiosis

Xinxin Xiong, Shenglei Feng, Xixiang Ma, Kuan Liu, Yiqian Gui, Bei Chen, Xu Fan, Fengli Wang, Xiaoli Wang and Shuiqiao Yuan**

Supporting Information

hnRNPC functions with HuR to regulate alternative splicing in an m6A-dependent manner and is essential for meiosis

Xinxin Xiong, et al.

Figure S1

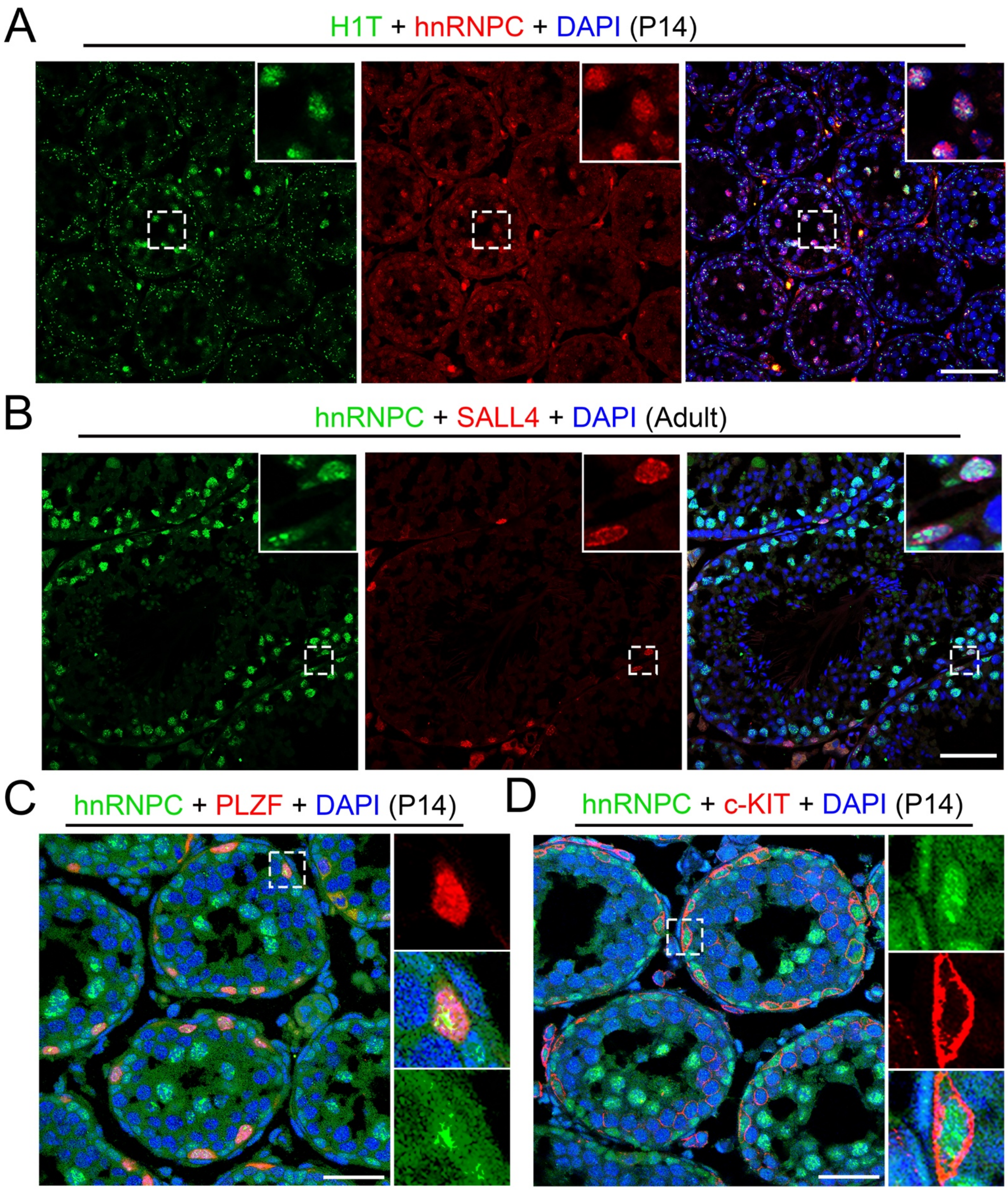

Figure S1. The expression pattern of hnRNPC during spermatogenesis. (A) Representative confocal images of immunofluorescence (IF) with anti-H1T antibody (green), anti-hnRNPC antibody (red) and DAPI (blue) on testis sections from postnatal day 14 (P14) wild-type (WT) mice are shown. Enlarged images are shown at top right. Scale bars = 50 μm. (**B)** Representative confocal images of IF with anti-hnRNPC antibody (green), anti-SALL4 antibody (red) and DAPI (blue) on testis sections from adult WT mice are shown. Enlarged images are shown at top right. Scale bars = 50 μm. **(C-D)** Representative confocal images of IF with anti-hnRNPC antibody (green), anti-PLZF (C)

or c-KIT (D) antibody (red) and DAPI (blue) on testis sections from P14 WT mice are shown. Enlarged right panels are shown for the circled square areas. Scale bars = 50 μm.

Figure S2

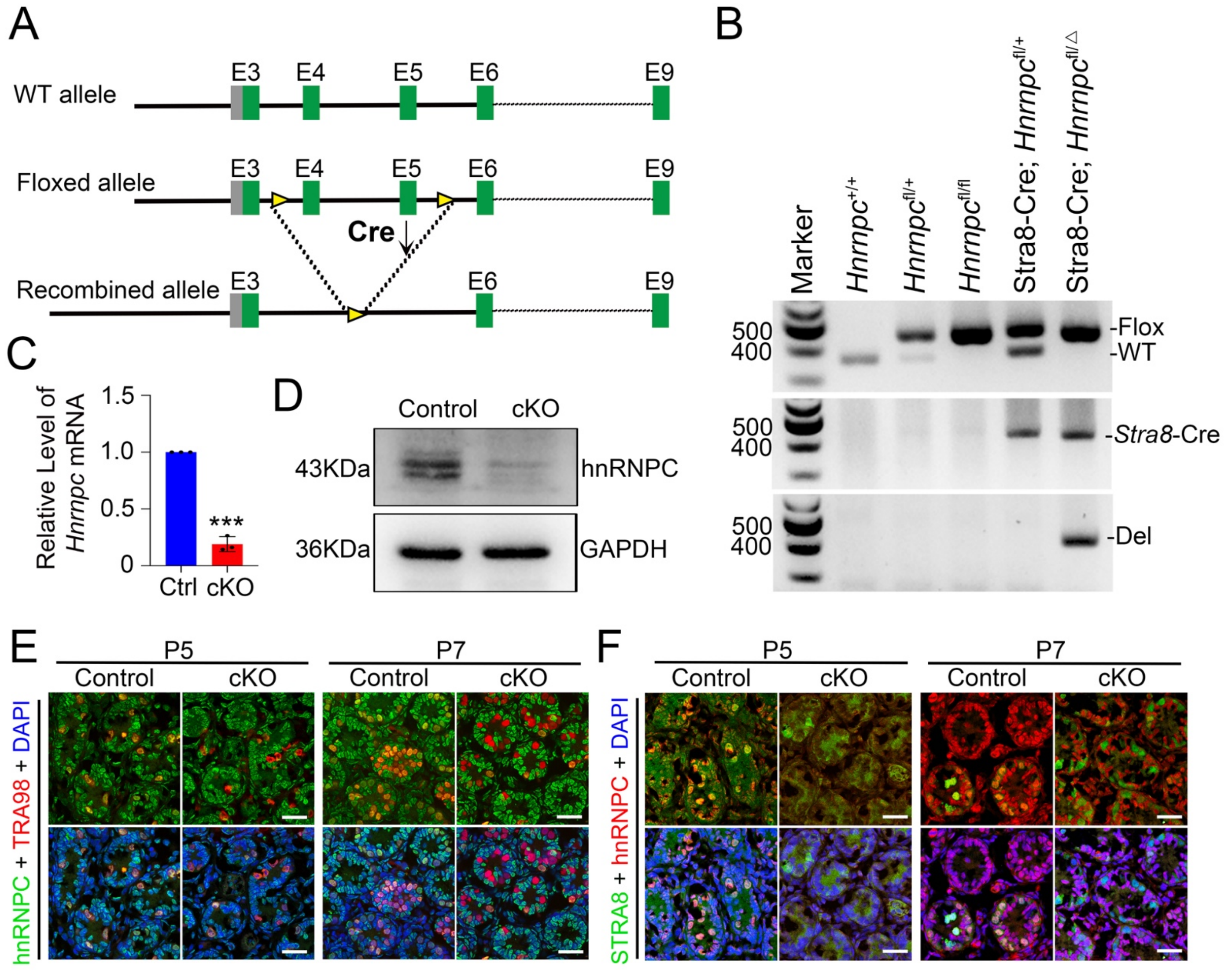

G

Male	Female	Number of pups
Stra8-Cre; *Hnrnpc*$^{fl/\triangle}$ 1#	WT	None
Hnrnpc$^{fl/\triangle}$ 1#	WT	7.2
Stra8-Cre; *Hnrnpc*$^{fl/\triangle}$ 2#	WT	None
Hnrnpc$^{fl/\triangle}$ 2#	WT	7.9
Stra8-Cre; *Hnrnpc*$^{fl/\triangle}$ 3#	WT	None
Hnrnpc$^{fl/\triangle}$ 3#	WT	7.6

Figure S2. Generation of germline conditional *Hnrnpc* knockout mouse models. (A) Genetic strategy used to generate hnRNPC cKO mice. LoxP sites were inserted to delete Exon 4 and 5 of *Hnrnpc* after Cre-mediated recombination. **(B)** Genotyping PCR of mouse tails was performed using

Hnrnpc and *Stra8*-Cre primers. **(C)** Real-time qPCR showing *Hnrnpc* mRNA levels in P8 control (Ctrl) and hnRNPC cKO (cKO) testes. Data are expressed as mean ± SD of at least three independent experiments. Two-tailed Student's *t*-test was used for statistical testing. ***$p < 0.001$. **(D)** Western blotting showing hnRNPC protein levels in adult control and cKO testes. GAPDH was used as a loading control. **(E)** Representative confocal images of IF with anti-hnRNPC antibody (green), anti-TRA98 antibody (red) and DAPI (blue) on testis sections from control and hnRNPC cKO mice at P5 and P7 are shown. Scale bars = 50 μm. **(F)** Representative confocal images of IF with anti-STRA8 antibody (green), anti-hnRNPC antibody (red) and DAPI (blue) on testis sections from control and hnRNPC cKO mice at P5 and P7 are shown. Scale bars = 50 μm. **(G)** Fertility test of adult control and hnRNPC cKO male mice.

Figure S3

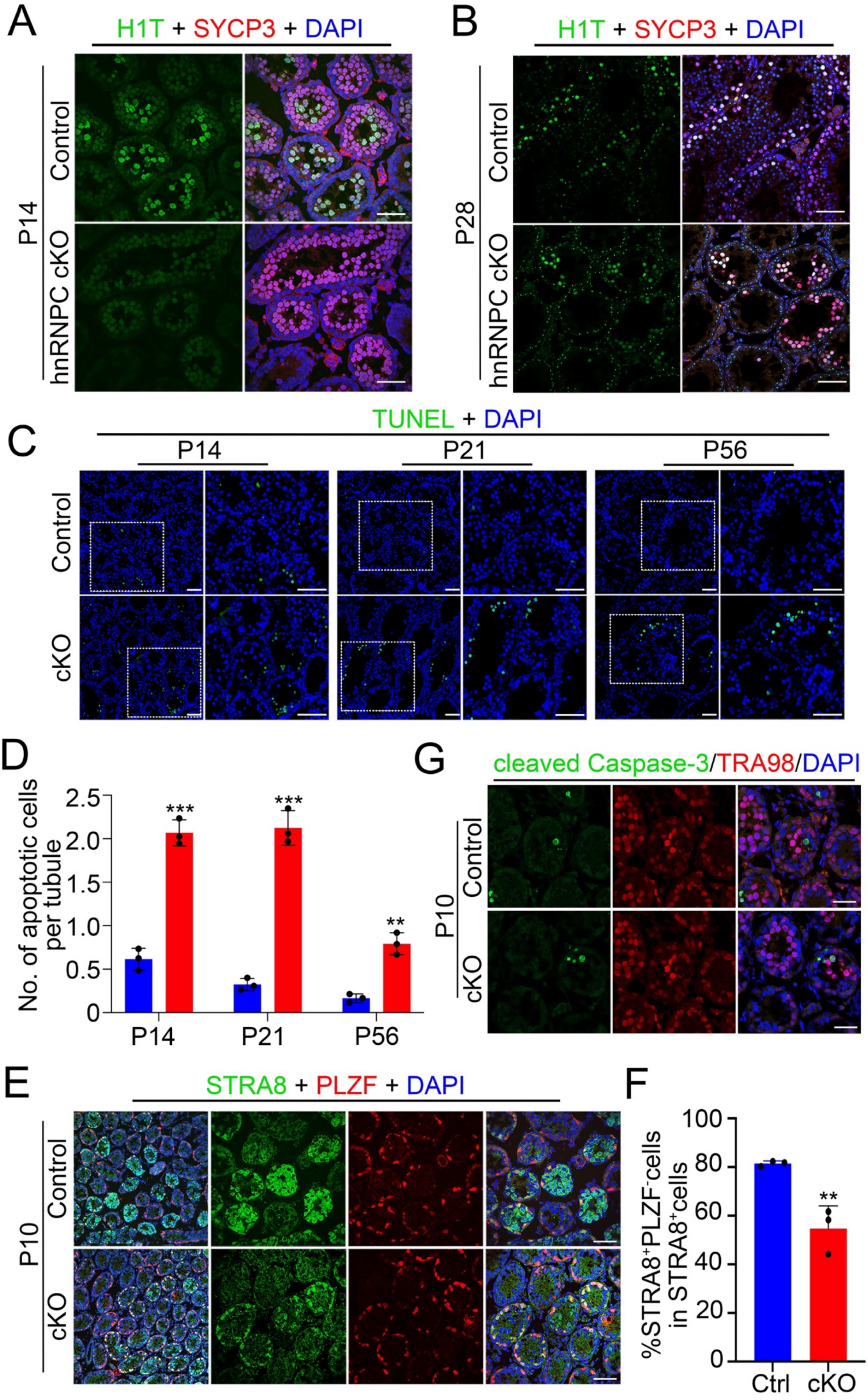
A
H1T + SYCP3 + DAPI
P14
Control
hnRNPC cKO
B
H1T + SYCP3 + DAPI
P28
Control
hnRNPC cKO
C
TUNEL + DAPI
P14
P21
P56
Control
cKO
D
No. of apoptotic cells per tubule
2.5
2.0
1.5
1.0
0.5
0
***
***
**
P14
P21
P56
G
cleaved Caspase-3/TRA98/DAPI
P10
Control
cKO
E
STRA8 + PLZF + DAPI
P10
Control
cKO
F
%STRA8+PLZF- cells in STRA8+ cells
100
80
60
40
20
0
**
Ctrl
cKO

Figure S3. Deletion of hnRNPC in germ cells results in defective meiosis initiation and spermatogonial development. (A-B) Representative confocal images of IF with anti-H1T antibody (green), anti-SYCP3 antibody (red) and DAPI (blue) on testis sections from p14 (A) or P28 (B) control and hnRNPC cKO mice are shown. Scale bars = 50 μm. **(C)** TUNEL staining (green) in control and hnRNPHC cKO mouse testes at P14, P21 and P56. DNA was stained with DAPI (blue). Enlarged images are shown in the right panels. Scale bars = 50μm. **(D)** The histogram shows the quantification of apoptotic cells per tubule (n = 3 per group) in (C). Data are presented as mean ± SD. $^{**}p < 0.01$, $^{***}p < 0.001$. **(E)** Representative confocal images of IF with anti-STRA8 antibody (green), anti-PLZF antibody (red) and DAPI (blue) on testis sections from control and hnRNPC cKO mice at P10 are shown. Scale bars = 50 μm. **(F)** Quantification of the ratio of STRA8^{+} PLZF^{-} cells to STRA8^{+} cells in (F). Data are presented as mean ± SD. n = 3 per group. Two-tailed Student's *t*-test was used for statistical testing. $^{**}p < 0.01$. **(G)** Representative confocal images of IF with anti-cleaved CASPASE3 antibody (green), anti-TRA98 antibody (red) and DAPI (blue) on testis sections from control and hnRNPC cKO mice at P10 are shown. Scale bars = 50 μm.

Figure S4

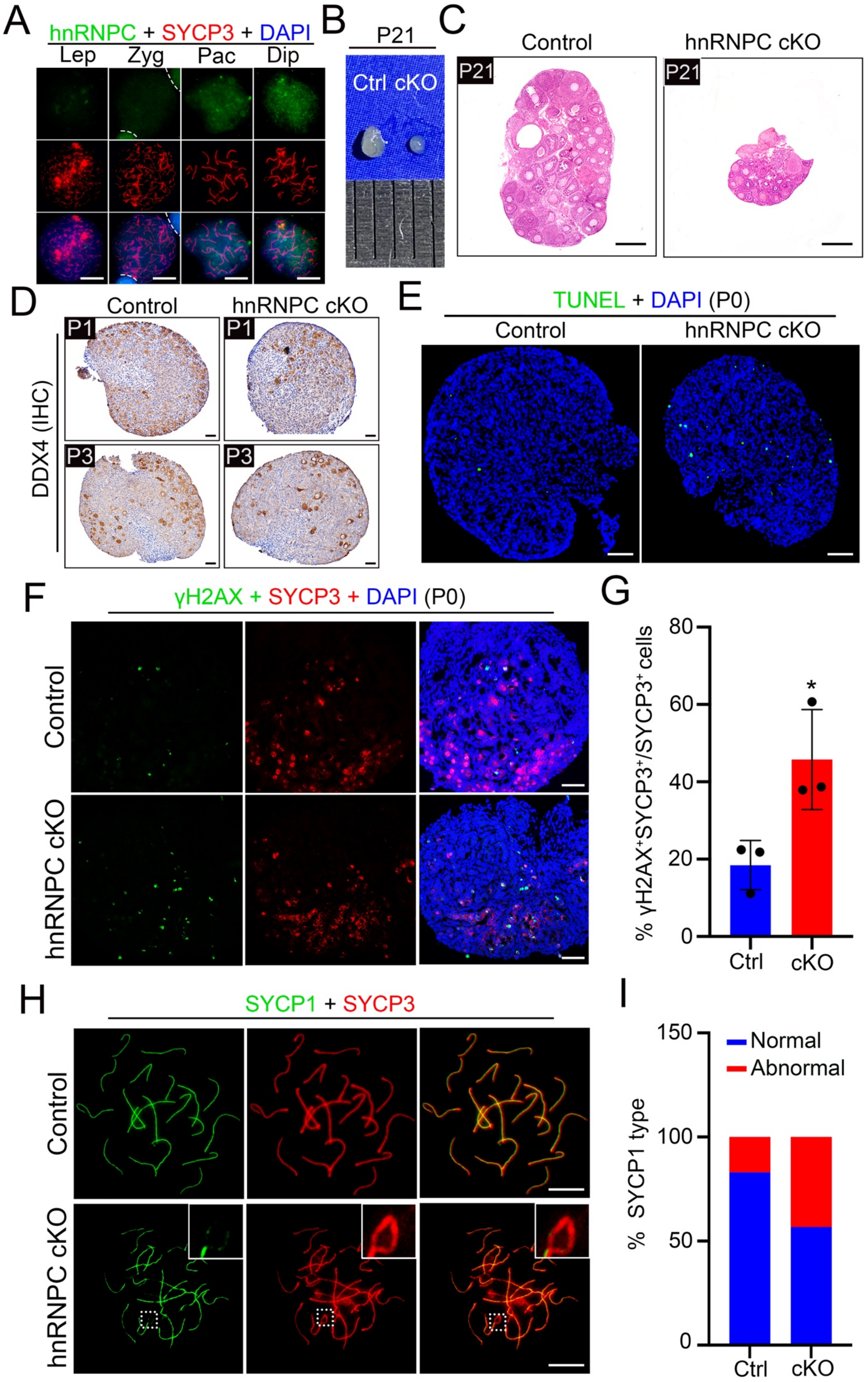
A
hnRNPC + SYCP3 + DAPI
Lep
Zyg
Pac
Dip
B
P21
Ctrl cKO
C
Control
hnRNPC cKO
P21
P21
D
Control
hnRNPC cKO
DDX4 (IHC)
P1
P1
P3
P3
E
TUNEL + DAPI (P0)
Control
hnRNPC cKO
F
γH2AX + SYCP3 + DAPI (P0)
Control
hnRNPC cKO
G
% γH2AX⁺SYCP3⁺/SYCP3⁺ cells
80
60
40
20
0
*
Ctrl
cKO
H
SYCP1 + SYCP3
Control
hnRNPC cKO
I
Normal
Abnormal
% SYCP1 type
150
100
50
0
Ctrl
cKO

Figure S4. hnRNPC is required for oogenesis. (A) Immunostaining with anti-hnRNPC antibody (green), anti-SYCP3 antibody (red) and DAPI (blue) on nuclear spreading oocytes from embryonic day 16.5 (E16.5) mice. Lep, leptotene; Zyg, zygotene; Pac, pachytene; Dip, diplotene Scale bars = 5 μm. **(B)** Gross morphology of ovaries harvested from P21 control and hnRNPC cKO mice. **(C)** Hematoxylin and eosin (H&E) staining on ovarian sections from P21 control and hnRNPC cKO ovaries. Scale bars = 50 μm. **(D**) Immunohistochemistry with anti-DDX4 antibody on ovarian sections from control and hnRNPC cKO mice at P1 and P3. Scale bars = 50 μm. **(E)** TUNEL staining in control and hnRNPC cKO ovaries at P0. Nuclei were counterstained with DAPI. Scale bars = 50 μm. **(F)** Representative confocal images of IF with anti-γH2AX antibody (green), anti-SYCP3 antibody (red) and DAPI (blue) on ovarian sections from P0 control and hnRNPC cKO mice are shown. Scale bars = 50 μm. **(G)** Quantification of the percentage of SYCP3 $^{+}$ γH2AX $^{+}$ cells in SYCP3^{+} cells in (F) (n = 3 mice per group). Data are presented as mean ± SD and two-tailed Student's t-test was used for statistical analysis. *$p < 0.05$. **(H)** Representative confocal images of IF with anti-SYCP1 antibody (green) and anti-SYCP3 antibody (red) on nuclear spreading oocytes from E16.5 control and hnRNPC cKO mice are shown. Enlarged images show chromosome asynapsis. Scale bars = 5 μm. **(I)** Quantification of the percentage of oocytes with normal and abnormal synapsis according to SYCP1 distribution in (H).

Figure S5

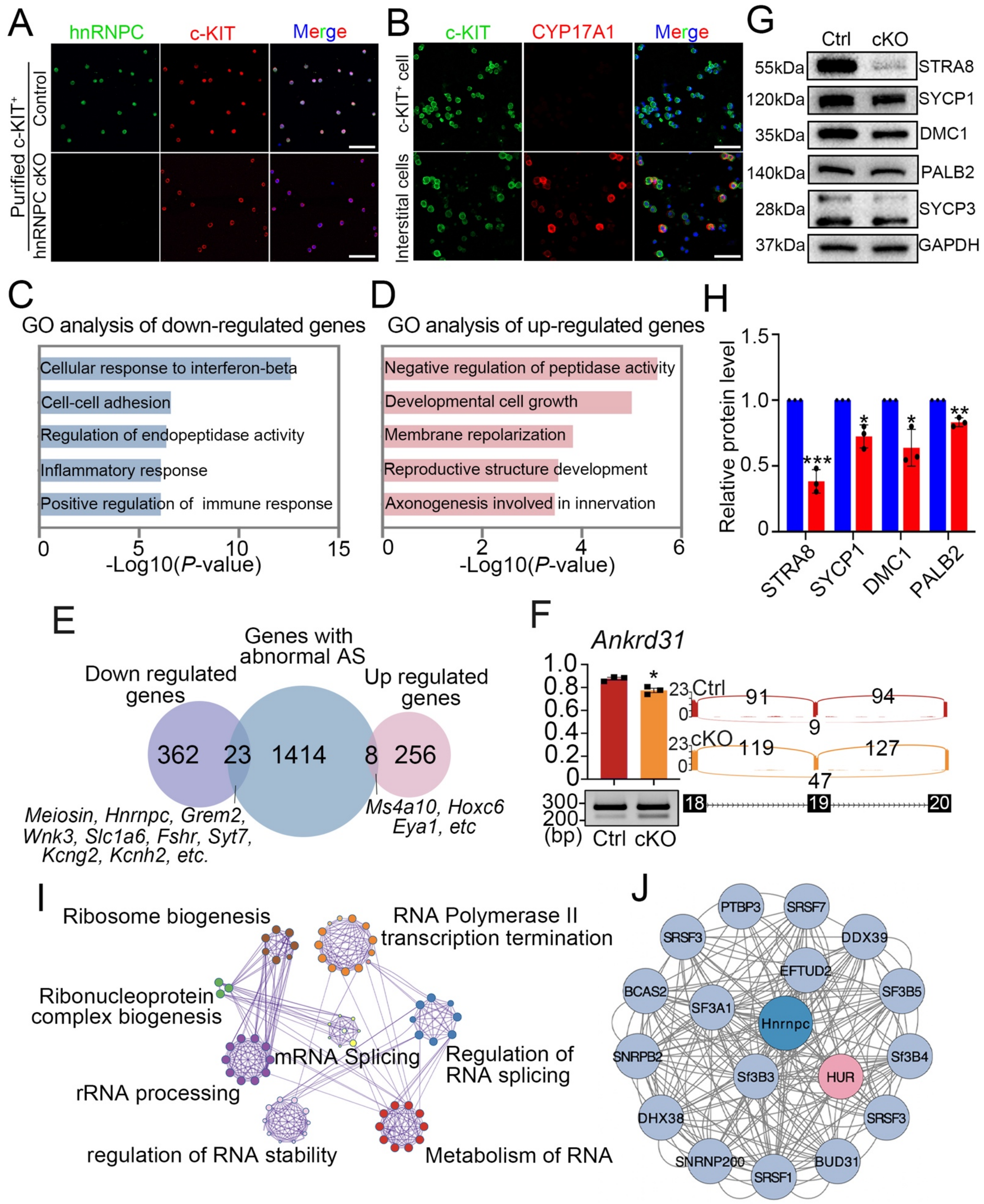

Figure S5. hnRNPC regulates alternative splicing. (A) Representative images of IF with anti-hnRNPC antibody (green), anti-c-KIT antibody (red) and DAPI (blue) on isolated differentiated spermatogonia from P8 control and hnRNPC cKO mice are shown. Scale bars = 50 μm. **(B)** Representative images of IF with anti-c-KIT antibody (green), anti-CYP17A1 antibody (red) and DAPI (blue) on isolated differentiated spermatogonia and interstitial cells are shown. Scale bars =

50 μm. **(C-D)** GO term analysis of the top 5 enriched biological processes of downregulated (C) or upregulated (D) genes determined by RNA-seq. **(E)** Venn diagram showing overlap between genes with abnormal alternative splicing (AS) and differentially expressed genes (DEGs) in hnRNPC cKO differentiated spermatogonia. **(F)** Quantification of the percent spliced in (PSI) value of *Ankrd31* derived from RNA-seq data and verified by RT-PCR. Visualization of *Ankrd31* is shown using the Integrative Genomics Viewer (IGV). Data are presented as mean ± SD, n = three biological replicates. A two-tailed Student's *t*-test was performed. $^{*}p < 0.05$. **(G)** Western blot analysis of protein levels of the selected genes with abnormal alternative splicing using germ cells from P10 control and hnRNPC cKO mice. GAPDH was used as a loading control. **(H)** The quantification of STRA8, SYCP1 and DMC1 was normalized to SYCP3 and PALB2 was normalized to GAPDH. Data are presented as the mean ± SD. n = 3. $^{*}p < 0.05$, $^{**}p < 0.01$, $^{***}p < 0.001$. **(I)** GO term enrichment analysis of hnRNPC interacting proteins identified by IP-MS. **(J)** The protein interaction network of 17 candidate proteins associated with mRNA splicing.

Figure S6

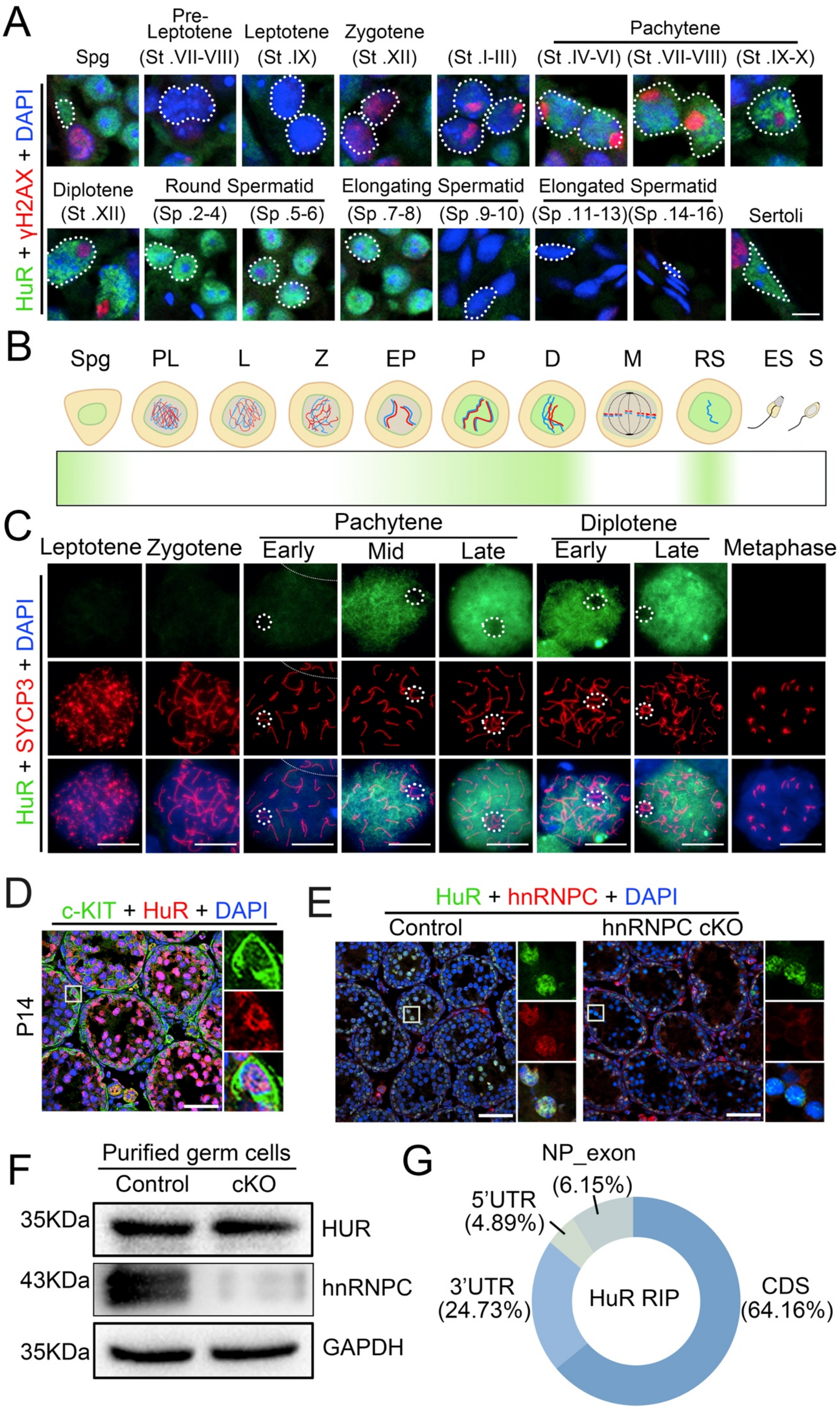
A
Spg
Pre-Leptotene (St .VII-VIII)
Leptotene (St .IX)
Zygotene (St .XII)
Pachytene
(St .I-III)
(St .IV-VI)
(St .VII-VIII)
(St .IX-X)
Diplotene (St .XII)
Round Spermatid
(Sp .2-4)
(Sp .5-6)
Elongating Spermatid
(Sp .7-8)
(Sp .9-10)
Elongated Spermatid
(Sp .11-13)
(Sp .14-16)
Sertoli
HuR + γH2AX + DAPI
B
Spg
PL
L
Z
EP
P
D
M
RS
ES
S
C
Leptotene
Zygotene
Pachytene
Early
Mid
Late
Diplotene
Early
Late
Metaphase
HuR + SYCP3 + DAPI
D
c-KIT + HuR + DAPI
P14
E
HuR + hnRNPC + DAPI
Control
hnRNPC cKO
F
Purified germ cells
Control
cKO
35KDa
HUR
43KDa
hnRNPC
35KDa
GAPDH
G
NP_exon (6.15%)
5'UTR (4.89%)
3'UTR (24.73%)
CDS (64.16%)
HuR RIP

Figure S6. HuR shows a dynamic expression pattern during spermatogenesis. (A) Representative confocal images of IF with anti-HuR antibody (green), anti-γH2AX antibody (red) and DAPI (blue) on testis sections from adult WT mice are shown. White dotted lines indicate the specific cell type as indicated. Scale bar = 10 μm. **(B)** Schematic diagram of hnRNPC expression during spermatogenesis. Spg: spermatogonia; PL, pre-leptotene; L, leptotene; Z, zygotene; EP, early pachytene; P, pachytene; D, diplotene; M, metaphase; RS, round spermatids; ES, elongating spermatids; S, spermatozoa. **(C)** Immunostaining with anti-HuR antibody (green), anti-SYCP3 antibody (red) and DAPI (blue) on nuclear spreading spermatocytes from adult WT mice. White circles indicate XY bodies. Scale bars = 5 μm. **(D)** Representative confocal images of IF with anti-c-KIT antibody (green), anti-HuR antibody (red) and DAPI (blue) on testis sections from control and WT mice at P14 are shown. Enlarged images are shown on the right. Scale bars = 50 μm. **(E)** Representative confocal images of IF with anti-HuR antibody (green), anti-hnRNPC antibody (red) and DAPI (blue) on testis sections from P14 control and hnRNPC cKO mice are shown. Enlarged images are shown on the right. The arrows indicate the hnRNPC KO cell in the hnRNPC cKO section. Scale bars = 50 μm. **(F)** Western blot analysis of HuR protein levels using germ cells from P10 control and hnRNPC cKO mice. GAPDH was used as a loading control. **(G)** The doughnut chart shows the distribution HuR binding peaks along the genome.

Figure S7

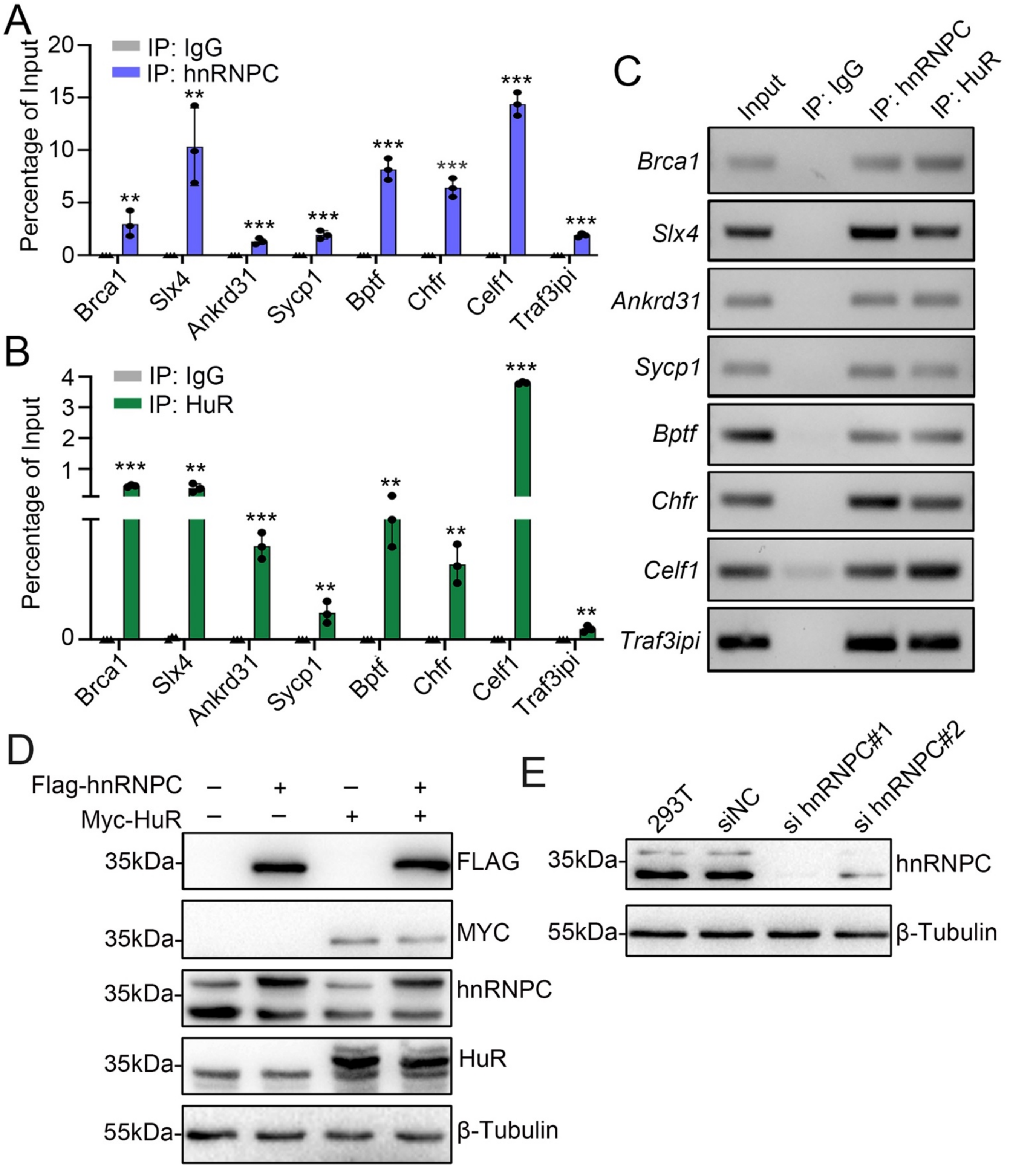

Figure S7. hnRNPC cooperates with HuR and has a similar binding capacity. (A-B) hnRNPC (A) or HuR (B) RIP-qPCR showing their binding ability to the transcripts of eight selected genes (*Brca1*, *Slx4*, *Ankrd31*, *Sycp1*, *Bptf*, *Chfr*, *Celfand*, and *Traf3ipi*) in purified c-KIT$^+$ germ cells. IgG was used as a negative control. Data are expressed as mean ± SD of at least three independent experiments. Two-tailed Student's t-test was used for statistical testing. ***p* < 0.01, ****p* < 0.001. **(C)** RIP-PCR verification for (A) and (B). **(D)** Immunoblot showing ectopic overexpression of Flag-hnRNPC and/or Myc-HuR in the HEK293 cell line. Both endogenous and ectopic protein levels are detected. **(E)** Immunoblot showing the knockdown efficiency of *Hnrnpc* using anti-hnRNPC and anti-β-tubulin antibodies.
